# CD14^+^ CD15^−^ HLA-DR^−^ myeloid-derived suppressor cells impair antimicrobial responses in patients with acute-on-chronic liver failure

**DOI:** 10.1136/gutjnl-2017-314184

**Published:** 2017-06-07

**Authors:** Christine Bernsmeier, Evangelos Triantafyllou, Robert Brenig, Fanny J Lebosse, Arjuna Singanayagam, Vishal C Patel, Oltin T Pop, Wafa Khamri, Rooshi Nathwani, Robert Tidswell, Christopher J Weston, David H Adams, Mark R Thursz, Julia A Wendon, Charalambos Gustav Antoniades

**Affiliations:** 1 Institute of Liver Studies, King’s College Hospital, King’s College London, London, UK; 2 Liver Biology Laboratory, Cantonal Hospital St. Gallen, St. Gallen, Switzerland; 3 Division of Digestive Diseases, St. Mary’s Campus, Imperial College London, London, UK; 4 Institute of Immunology and Immunotherapy, NIHR Biomedical Research Unit, Centre for Liver Research, University of Birmingham, Birmingham, UK

**Keywords:** ACLF, ALF, cirrhosis, MDSC, immune suppression, bacterial infection

## Abstract

**Objective:**

Immune paresis in patients with acute-on-chronic liver failure (ACLF) accounts for infection susceptibility and increased mortality. Immunosuppressive mononuclear CD14^+^HLA-DR^−^ myeloid-derived suppressor cells (M-MDSCs) have recently been identified to quell antimicrobial responses in immune-mediated diseases. We sought to delineate the function and derivation of M-MDSC in patients with ACLF, and explore potential targets to augment antimicrobial responses.

**Design:**

Patients with ACLF (n=41) were compared with healthy subjects (n=25) and patients with cirrhosis (n=22) or acute liver failure (n=30). CD14^+^CD15^−^CD11b^+^HLA-DR^−^ cells were identified as per definition of M-MDSC and detailed immunophenotypic analyses were performed. Suppression of T cell activation was assessed by mixed lymphocyte reaction. Assessment of innate immune function included cytokine expression in response to Toll-like receptor (TLR-2, TLR-4 and TLR-9) stimulation and phagocytosis assays using flow cytometry and live cell imaging-based techniques.

**Results:**

Circulating CD14^+^CD15^−^CD11b^+^HLA-DR^−^ M-MDSCs were markedly expanded in patients with ACLF (55% of CD14+ cells). M-MDSC displayed immunosuppressive properties, significantly decreasing T cell proliferation (p=0.01), producing less tumour necrosis factor-alpha/interleukin-6 in response to TLR stimulation (all p<0.01), and reduced bacterial uptake of *Escherichia coli* (p<0.001). Persistently low expression of HLA-DR during disease evolution was linked to secondary infection and 28-day mortality. Recurrent TLR-2 and TLR-4 stimulation expanded M-MDSC in vitro. By contrast, TLR-3 agonism reconstituted HLA-DR expression and innate immune function ex vivo.

**Conclusion:**

Immunosuppressive CD14^+^HLA-DR^−^ M-MDSCs are expanded in patients with ACLF. They were depicted by suppressing T cell function, attenuated antimicrobial innate immune responses, linked to secondary infection, disease severity and prognosis. TLR-3 agonism reversed M-MDSC expansion and innate immune function and merits further evaluation as potential immunotherapeutic agent.

**Significance of this study**

**What is already known on this subject?**

- Immune paresis has been described in patients with acute-on-chronic liver failure (ACLF) and is postulated to be responsible for infection susceptibility and adverse outcome.
- Low HLA-DR expression on circulating monocytes is associated with poor prognosis.
- Myeloid-derived suppressor cells (M-MDSCs) have recently been defined as CD14^+^CD15^−^CD11b^+^HLA-DR^−^ cells with T cell suppressive function and have been identified to dampen immune responses in sterile and malignant inflammatory diseases.

**What are the new findings?**

- M-MDSCs are expanded in the circulation in patients with acute liver failure and ACLF.
- Activation of systemic inflammatory response syndrome and circulating pathogen-associated molecular patterns trigger the expansion of M-MDSC in patients with ACLF.
- In addition to suppressing T cell activation, M-MDSCs suppress pathogen uptake and Toll-like receptor (TLR)-elicited proinflammatory responses to microbial challenge.
- Persistence of the M-MDSC phenotype during disease progression confers a poor outcome and is associated with an increase incidence of infections.
- Proof-of-principle data indicate that proportions of suppressive M-MDSC can be reduced and their antimicrobial function augmented in vitro following administration of TLR-3 agonist polyI:C

**How might it impact on clinical practice in the foreseeable future?**

- M-MDSC may represent mechanistic biomarkers of impaired antimicrobial responses and infection susceptibility in patients with cirrhosis.
- TLR-3 agonism requires further evaluation as an immunotherapeutic strategy to improve antimicrobial responses in patients with ACLF.

## Introduction

Acute-on-chronic liver failure (ACLF) is characterised as high morbidity and mortality due to profound activation of systemic inflammatory response syndrome (SIRS) and development of multiple-organ dysfunction.^1^ Recent reports indicate that activation of SIRS responses in ACLF results in immune dysregulation and is postulated to be responsible for defective immune responses to microbial cues, termed immune paresis.^2 3^ Immune paresis is associated with increased frequency of infectious events in cirrhosis^4^ and frequently leads to acute decompensation, extrahepatic organ failure and increased mortality.^5^ Development of secondary bacterial infections in patients with ACLF is associated with a 30-day mortality of 49% and is highly predictive of adverse outcome.^4^

Over the previous decade, it has emerged that myeloid-derived suppressor cells (MDSCs) are elicited under various pathological conditions. MDSCs were initially defined in murine cells expressing CD11b and Gr-1 antigen, defined by their ability to suppress T cell proliferative and antitumour responses. However, human MDSCs are a heterogeneous population including a CD14^–^CD11b^+^CD33^+^CD15^+^ polymorphonuclear fraction (PMN-MDSC) and a CD11b+CD14^+^HLA-DR^low/–^ mononuclear fraction (M-MDSC).^6–8^ M-MDSCs have recently been identified in a wide number of hepatic (eg, chronic viral infection, hepatocellular cancer),^9–11^ non-hepatic systemic (eg, sepsis),^12 13^ organ-specific inflammatory diseases^14 15^ and malignancies.^16 17^ The increased frequency of this suppressive immune cell population has been associated with impaired T cell responses and serves as an immunological biomarker of disease severity and outcome.

Although the ability of MDSC to supress T cell responses has been extensively documented, their innate immune and antimicrobial responses remain relatively unexplored. Furthermore, while the existence of a HLA-DR^low/−^ monocyte population has been described in acute hepatic inflammatory disorders,^18–21^ little is known about their lineage, innate and adaptive immune function, and their candidacy as a potential immunotherapeutic target to restore antimicrobial responses in patients with ACLF.

We hypothesised that persistent exposure to pathogen-associated molecular patterns (PAMPs), activation of SIRS and the development of ACLF trigger the expansion of immunosuppressive M-MDSCs that serve to impair both innate and adaptive responses to microbial agents thereby contributing to the increased frequency of infections encountered in patients with ACLF. We therefore sought to delineate the presence and function of M-MDSC in patients with ACLF, and explore potential targets to modulate their function in order to augment antimicrobial responses.

## Materials and methods

### Patients and sampling

From January 2013 to June 2015, we consecutively recruited patients admitted to King’s College Hospital for stable cirrhosis (n=22), ACLF (n=41) or acute liver failure (ALF; n=30). Patients with ACLF fulfilled the established diagnostic criteria developed by the European Association for the Study of the Liver-CLIF (Consortium on Chronic Liver Failure).^1^ Cirrhosis was diagnosed by a previous liver biopsy or clinical presentation with typical ultrasound or CT imaging. Exclusion criteria were age younger than 18 years, malignancy and immunosuppressive therapy other than corticosteroids. Within 24 hours of admission to hospital, blood was sampled for ex vivo analysis of monocyte differentiation and function. Results were compared with healthy subjects (n=25). For patients with ALF and ACLF, sequential tests were done on days 3, 5, 7 and 14 after admission when feasible. Power calculations indicate that in order to detect significant differences in immune function and phenotype (eg, tumour necrosis factor-alpha (TNF-α) secretion) with 80% statistical power at the 5% significance level, a minimum of 28 patients would need to be recruited into the ACLF and ALF study groups.

Routinely assessed full blood count, international normalised ratio, liver and renal function tests, lactate and clinical variables were entered prospectively into a database. The following disease severity scores were calculated: Child-Pugh, Model for End-Stage Liver Disease (MELD), CLIF-SOFA (Sequential Organ Failure Assessment),^1^ North American Consortium for Study of End-stage Liver Disease (NACSELD),^22^ Acute Physiology and Chronic Health Evaluation II, Simplified Acute Physiology Score II, SOFA scores; infections and 28-day survival were documented. The study had been approved by the King’s College Hospital Ethics Committee (12/LO/0167). Assent was obtained by the patients’ nominated next of kin if they were unable to provide informed consent themselves.

### CD14+ cell isolation

CD14+ monocytic cells were isolated from peripheral blood mononuclear cells (PBMCs) using the CD14 Microbeads or Pan-Monocyte Isolation Kit (Miltenyi Biotec, Bergisch Gladbach, Germany) as previously described.^23^ Purity of monocytes was assessed by flow cytometry.

### Flow cytometry-based phenotyping of monocytes and assessment of cytokine responses to Toll-like receptor stimulation

Phenotyping of monocytes and measurement of inflammatory responses to Toll-like receptor (TLR) stimulation were done based on flow cytometry as previously described.^23^ Monoclonal antibodies against CD14, CD16, CD86, CD163, CD64, CD11b, chemokine receptor (CCR) 2, CCR5, CCR7, Annexin V, 7-AAD (BD Biosciences, Oxford, UK); HLA-DR, CD32, CX3CR1 (eBioscience, Hatfield, UK), and hMer (R&D Systems, Abingdon, UK) were purchased from the indicated companies. Results are expressed as the percentage of positive cells or mean fluorescence intensity (MFI). TNF-α and interleukin-6 (IL-6; BD Biosciences) levels were determined after a 4–6 hour incubation of PBMCs with lipopolysaccharide (LPS; 100 ng/mL), Pam3CSK4 (5 µg/mL), CpG-ODN2006 (10 µg/mL) (Invivogen, San Diego, USA). Flow cytometry data were analysed using Flowlogic (Inivai Technologies, Mentone, Australia) or FlowJo software (V.10.2; Ashland, Oregon, USA).

### Mixed lymphocyte reaction

CD14+ monocytic cells from a healthy donor were isolated using the Pan-Monocyte Isolation Kit (Miltenyi Biotec) as previously described.^23^ Cells were cultured in 25% plasma from patients with ACLF (n=8) or healthy controls (n=2) for 16 hours and cocultured with allogeneic CD3+ T cells from a different healthy donor, isolated using the Pan-T-cell Isolation Kit (Miltenyi Biotec) in a 1:1 ratio. T cell stimulation was induced with anti-CD2/CD3/CD28 beads (T cell Activation/Expansion Kit; Miltenyi Biotec) according to the manufacturer’s protocol (2.5×10^5^ cells in 250 µL of Roswell Park Memorial Institute medium, 5% AB serum (Sigma-Aldrich, Gillingham, Dorset, UK), 1% penicillin/streptomycin). Cells were stained with carboxyfluorescein succinimidyl ester at day 0. Proliferation was assessed at day 3 of coculture. Experiments were performed in triplicate.

### Whole blood phagocytosis assay

The pHrodo *Escherichia coli* Red and *Staphylococcus aureus* Green BioParticles Phagocytosis Kit for Flow Cytometry was purchased from Invitrogen, Paisley, UK. The instructions of the manufacturer were precisely followed. CD14+ cells were stained with antibodies against CD14 (APC-H7), CD16 (PercP-Cy5.5), HLA-DR (APC), and CD3, CD15, CD19, CD56 (all fluorescein isothiocyanate [FITC] channel) for 20 min at 4°C. Cells were acquired on BD FACS CANTO II flow cytometer (see online supplementary methods).

10.1136/gutjnl-2017-314184.supp7 Supplementary Methods

### Ex vivo phagocytosis assay in PBMC

For the assessment of phagocytosis of PBMCs in vitro, 500 000 healthy PBMCs per well were cultured (24 hours) in complete medium containing 20% healthy controls or ACLF plasma. Cells were stimulated with various agents (polyI:C 10 µg/mL, LPS 1 and 10 µg/mL, CpG-ODN2006 1 and 10 µg/mL) for 4 hours. Harvested cells were supplemented by 10% autologous plasma and pHrodo *E. coli* Red BioParticles (Invitrogen) were added for 60 min (see online supplementary methods).

### Phagocytosis of CD14+ cells: Cell-IQ

CD14+ cells were isolated and cultured (24 hours) in complete media with 25% plasma from two healthy subjects and two patients with ACLF. Cells were supplemented with 10% human AB serum and pHrodo *E. coli* Red BioParticles (Invitrogen).

Real-time cell imaging of the uptake of BioParticles was captured by a Cell-IQ system (CMTechnologies), running Imagen software V.2.8.12.0 and analyser V.3.3.0 (see online supplementary methods).

### Quantification of bacterial 16S rDNA levels in whole blood using TaqMan qRT-PCR

The procedure was carried out under strict aseptic conditions as previously reported^24^ (see online supplementary methods).

### In vitro models for the generation of M-MDSC-like cells

The models were adapted from Pena *et al*.^25^ 9×10^6^ PBMCs per well were cultured on a 12-well plate in 2000 µL X-Vivo medium (Lonza, Basel, Switzerland) in a 37°C, 5% CO_2_ environment. Cells were stimulated with or without LPS 10 ng/mL, Pam3CSK4 5 µg/mL (Invivogen) or a combination of LPS and Pam3CSK4, respectively, for 24 hours. After this time, cells were rechallenged with LPS or Pam3CSK4 for a further 4 hours, and then subjected to diverse experiments such as intracellular staining of cytokine production, immunophenotyping, phagocytosis and viability assays as described above.

For the plasma experiments, CD14+ cells were cultured (16 hours) in plasma from healthy subjects (n=3) and patients with cirrhosis (n=3) and ACLF (n=6), respectively. Subsequently, the M-MDSC subset was identified and phenotyped as described above. Parts of the cells were transferred to fresh medium, stimulated with LPS 100 µg/mL (5 hours) and supernatants were used for S100A8/A9 ELISA (Systems).

### Phagocytosis PCR array

The RT^2^ Profiler phagocytosis PCR array (Cat No 330231 PAHS-173ZA) was purchased from SABiosciences, Quiagen, Manchester, UK. The process from RNA extraction to cDNA synthesis and quantitative RT-PCR was performed exactly as per manufacturer’s protocol (see online supplementary methods).

### Statistical analyses

Data are expressed as the median/IQR unless otherwise specified. For data that did not follow a normal distribution, the significance of differences was tested using the Mann-Whitney or Wilcoxon tests; Spearman correlation coefficients and area under the receiver operating characteristic curve (AUROC) were calculated. Graphs were drawn using Prism 7.0a (GraphPad, La Jolla, California).

## Results

### Patient characteristics

In comparison to patients with ACLF (n=41), patients with stable cirrhosis (n=22) and ALF (n=30) were included. Clinical characteristics of the different groups including disease stratification scores, the evidence of infection and prognosis are summarised in table 1. A 28-day transplant-free survival was 39% in the ACLF group and 56.6% in the ALF group, while 17.1% and 23.3% were transplanted, 43.9% and 20% died, respectively.

**Table 1:** Clinical characteristics of the cohort including patients with cirrhosis, ACLF and ALF

Parameter	Cirrhosis	ACLF	ALF
	(n=22)	(n=41)	(n=30)
Age (years)	55 (46–62)	48 (38–60)*	35 (27–46)
Child-Pugh	8 (6–9)	12 (11–13)*†	13 (11–13)
MELD	10 (9–15)	31 (22–39)*†	40 (33.5–40)
CLIF-SOFA	3.5 (2–5)	13 (11–16)†	—
NACSELD	0	2 (1–3)†	—
SOFA	NA	13 (12–14)	13 (11–16)
APACHE II	NA	22 (19–26)	20 (13–24)
SAPS II	NA	36 (27–41.5)	40 (28–50)
Transplantation, % (n)	0%	17.1% (7)	23.3% (7)
28-day mortality, % (n)	0%	43.9% (18)	20% (6)
28-day transplant-free survival, % (n)	100%	39% (16)	56.6% (17)
Culture-positive infection at admission, % (n)	0% (0)	9.8% (4)	6.6% (2)
Secondary infectious complications, % (n)	5% (1)	26.8% (11)	16.6% (5)
Bacterial DNA at admission (pg/mL)	3.5 (0.8)	6.7 (5.9)†	NA
Antibiotic treatment at admission, % (n)	5% (1)	87.8% (36)	96.6% (29)
Bilirubin (μmol/L)	27 (19–52)	152 (83–284)*†	83 (49–169)
Albumin (g/L)	34 (31–37)	24 (21–28)*†	25 (24–29)
INR	1.3 (1.2–1.5)	2.0 (1.7–2.7)*†	4.0 (2.74–7.27)
ASAT (U/L)	47 (31–66)	115 (69–288)*†	5250 (1329–7286)
Lactate (mmol/L)	NA	1.8 (1.2–2.8)*	3 (2.2–4.9)
WBC (×10^9^/L)	4.84 (3.45–7.36)	9.82 (6.25–14.37)†	10.97 (8.28–15.81)
Neutrophils	3.03 (2.03–4.92)	7.59 (4.69–12.11)†	8.81 (6.8–14.79)
Monocytes (x10^9^/L)	0.42 (0.3–0.58)	0.51 (0.33–0.88)*	0.25 (0.16–0.51)
CRP (mg/dL)	8.8 (5.7–22.4)	50.5 (23.1–79.2)*†	19.3 (7.4–40.5)
SIRS score	0 (0–1)	2 (1–2)†	2 (1–3)

All data are presented as median (IQR), unless otherwise specified in the corresponding rows.

*Significant difference comparing ACLF to ALF.

†Significant difference comparing ACLF to cirrhosis; p<0.05, Mann-Whitney U tests.

ACLF, acute-on-chronic liver failure; ALF, acute liver failure; APACHE II, Acute Physiology and Chronic Health Evaluation II; ASAT, aspartate aminotransaminase; CLIF, Consortium on Chronic Liver Failure; CRP, C-reactive protein; INR, international normalised ratio; MELD, Model for End-Stage Liver Disease; NA, not applicable; NACSELD, North American Consortium for Study of End-stage Liver Disease; SAPS II, Simplified Acute Physiology Score II; SIRS, systemic inflammatory response syndrome; SOFA, Sequential Organ Failure Assessment score; WBC, white blood cells.

Culture-positive infectious complications occurred in 36.6% (n=15) of patients with ACLF, while n=4 had been admitted with primary infections and n=12 developed secondary infections as previously defined by Bajaj *et al*,^4^ and in 23% (n=7) of patients with ALF (primary infections n=2, secondary infections n=5) (table 1). Infectious complications were caused by gram-negative pathogens (*E. coli*, *Pseudomonas aeruginosa*, *Acinetobacter baumannii*, *Klebsiella pneumoniae*, *Serratia marcescens*), gram-positive pathogens (*S. aureus*, *S. epidermidis*, *Enterococcus faecium*, *Streptococcus anginosus*, *Clostridium difficile*), atypical bacteria (*Mycoplasma pneumoniae*) and fungi (*Candida albicans*, *C. tropicalis*).

### Increased proportions of circulating immunosuppressive monocytic myeloid-derived suppressor cells in ACLF

**Figure 1:**
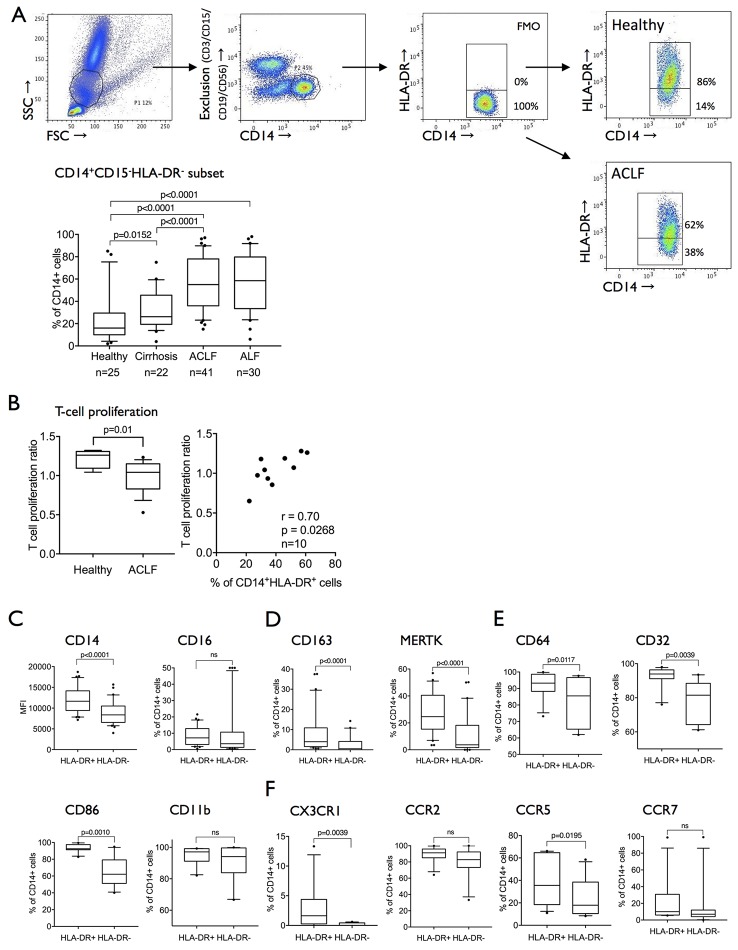
The expanded CD14^+^CD15^−^HLA-DR^−^ population in ACLF is attributed to the previously defined monocytic MDSC subset. (A) Gating strategy to determine CD14^+^CD15^−^HLA-DR^−^ cells: whole blood was sorted, monocytic cells were selected, and non-monocytic cells were excluded by lineage gating (CD3, CD15, CD19, CD56). CD14+, lineage negative cells were divided into HLA-DR+ and HLA-DR- subsets using FMO. Prevalences of the CD14^+^CD15^−^HLA-DR^−^ populations in healthy subjects, patients with cirrhosis, ACLF and ALF. (B) T cell proliferation in a mixed lymphocyte reaction assay is significantly reduced in ACLF and in relation to HLA-DR expression on monocytes in coculture (healthy n=2, ACLF n=8). (C**–**F). A large panel of myeloid cell differentiation markers was compared between the CD14^+^HLA-DR^+^ and CD14^+^HLA-DR^−^ populations. The CD14^+^HLA-DR^−^ monocytic subset appears to be CD14^+^CD15^−^CD11b^+^HLA-DR^−^CD64^+^ in line with the previously defined monocytic MDSC (M-MDSC). (C) Classical markers of monocyte differentiation were reduced in M-MDSC (CD14, CD163, MERTK). (D) FCγ receptors (CD64, CD32) were abundantly expressed in M-MDSC but lower in comparison to CD14^+^HLA-DR^+^ cells (n=30) and (E) markers of monocyte activation (CD86, CD11b) were reduced or equally expressed in M-MDSC. (F) Selected chemokine receptor expression (CX3CR1, CCR5) was lower in the M-MDSC population (n=11). Data are expressed as MFI and % of CD14+ cells and shown as box plots; Wilcoxon tests. ACLF, acute-on-chronic liver failure; ALF, acute liver failure; FMO, fluorescence minus one control; FSC, forward scatter; MDSC, myeloid-derived suppressor cells; MERTK, Mer Tyrosine Kinase; MFI, mean fluorescence intensity; SSC, side scatter.

Reduced expression of HLA-DR in circulating monocytes from patients with liver failure^18–21^ and other systemic inflammatory pathologies has been previously reported and correlated with adverse outcome. Recent advances have identified the existence of an HLA-DR^low/neg^ (CD14+CD15-CD11b+CD64+) expressing myeloid cell population with immunosuppressive capabilities termed monocytic myeloid-derived suppressor cells (M-MDSCs).^6 8 16^ In line with recent phenotypic and functional classification of M-MDSC,^8^ we show a marked expansion of CD14^+^HLA-DR^−^CD15^−^CD11b^+^CD64^+^ myeloid cells in ACLF (55% (median; IQR 36%–78%)) and ALF (58.5% (median; IQR 34%–80%)) when compared with patients with stable cirrhosis and healthy subjects (figure 1A) which have a suppressive effect on T cell proliferation when tested in an allogeneic mixed leucocyte reaction (figure 1B).

In comparison to CD14+HLA-DR+ cells, CD14+HLA-DR− cells have reduced expression of differentiation/scavenger (CD163, MERTK), activation/costimulatory (CD86), phagocytosis (CD64, CD32), certain homing markers (CX3CR1, CCR5) but similar expression of myeloid lineage marker CD11b and CCR2 (figure 1C–F). Importantly, this immune cell subset detailed here as M-MDSCs markedly differs from the recently identified suppressive MERTK+ population which is characterised by high expression of differentiation and activation markers such as CD163 and HLA-DR.^23^

### Circulating M-MDSCs in ACLF have impaired innate responses to microbial challenge and refer to poor prognosis and infection susceptibility

**Figure 2:**
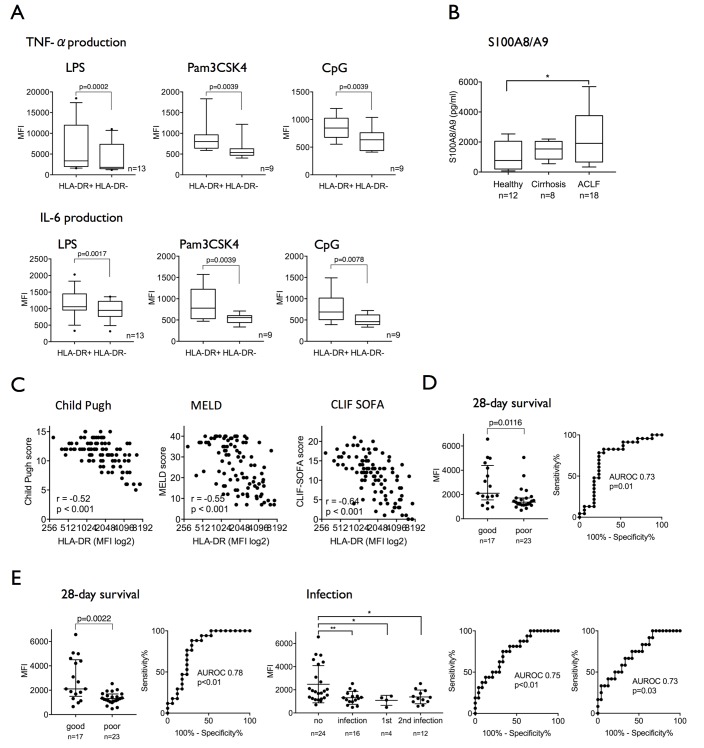
The CD14^+^HLA-DR^−^ M-MDSC population in ACLF is immunosuppressive and refers to poor prognosis and infection susceptibility. (A) TNF-α and IL-6 production in response to endotoxin stimuli (LPS/TLR-4, n=13; Pam3CSK4/TLR-2, CpG/TLR-9, n=9) ex vivo is significantly reduced in M-MDSC compared with HLA-DR+ monocytes. (B) S100A8/A9 protein levels are significantly elevated in plasma from patients with ACLF. (C) HLA-DR expression on monocytes in patients with ALCF and cirrhosis strongly correlated with validated disease severity scores (Child-Pugh, MELD, CLIF-SOFA; n=98). (D) Admission HLA-DR expression predicted 28-day survival (sensitivity 88%, specificity 77%, criterion MFI >1758). (E) Median HLA-DR expression over 14 days following admission predicted 28-day survival (sensitivity 74%, specificity 77%, criterion MFI >1617) and the presence/development of infection (left AUROC: 75% and a specificity of 67% for the criterion MFI <1500) as well as the onset of secondary infection alone (right AUROC: sensitivity 67%, specificity 67%, criterion MFI <1488). ACLF, acute-on-chronic liver failure; AUROC, area under the receiver operating characteristic curve; CLIF, Consortium on Chronic Liver Failure; IL-6, interleukin-6; LPS, lipopolysaccharide; MELD, Model for End-Stage Liver Disease; MFI, mean fluorescence intensity; M-MDSC, monocytic myeloid-derived suppressor cells; SOFA, Sequential Organ Failure Assessment score; TLR, Toll-like receptor; TNF-α, tumour necrosis factor-alpha.

Compared with healthy subjects, CD14+ cells from patients with ACLF exhibit attenuated proinflammatory responses (TNF-α/IL-6) to TLR-2 (Pam3CSK4), TLR-4 (LPS) and TLR-9 (CpG) challenge indicating reduced innate responses to several pathogen-induced TLR signalling pathways (see online supplementary figure 1). Compared with classical CD14+HLA-DR+ monocytes, M-MDSCs in ACLF were characterised by defective proinflammatory cytokine production in response to all different TLR ligands (figure 2A). Furthermore, S100A8/A9 protein levels, an inflammatory mediator synthesised and secreted preferentially by M-MDSC,^26^ were significantly higher in the plasma of patients with ACLF compared with healthy subjects and patients with cirrhosis (figure 2B).

10.1136/gutjnl-2017-314184.supp1 Supplementary Figure 1

In our cohort of patients with cirrhosis and ACLF, HLA-DR expression on CD14+ myeloid cells strongly negatively correlated with established scores of liver disease severity Child-Pugh, MELD, CLIF-SOFA (figure 2C), NACSELD score (r=−0.53, p<0.001, n=98) and also SIRS score (r=−0.46, p<0.001, n=96). Moreover, admission HLA-DR expression on CD14+ cells predicted 28-day survival with a sensitivity of 78% and a specificity of 76% for the criterion MFI >1758 (figure 2D).

Median HLA-DR expression over the first 14 days following admission predicted 28-day survival with a sensitivity of 74% and a specificity of 77% for the criterion MFI >1617 and the onset of infection within 28 days of hospitalisation with a sensitivity of 75% and a specificity of 67% for the criterion MFI <1500. Furthermore, it predicted both the presence of primary infection (AUROC 0.82/p=0.04, sensitivity 75%, specificity 71%, criterion MFI <1346; data not shown) and the development of secondary infection (AUROC 0.73/p=0.03, sensitivity 67%, specificity 67%, criterion MFI <1488) (figure 2E). These observations corroborate previous reports linking HLA-DR expression on monocytes with adverse prognosis in patients with ACLF^20 21^ and newly suggest an association between the persistence of the M-MDSC population during the course of disease and infection susceptibility.

### M-MDSCs display impaired bacterial uptake and clearance in ACLF

**Figure 3:**
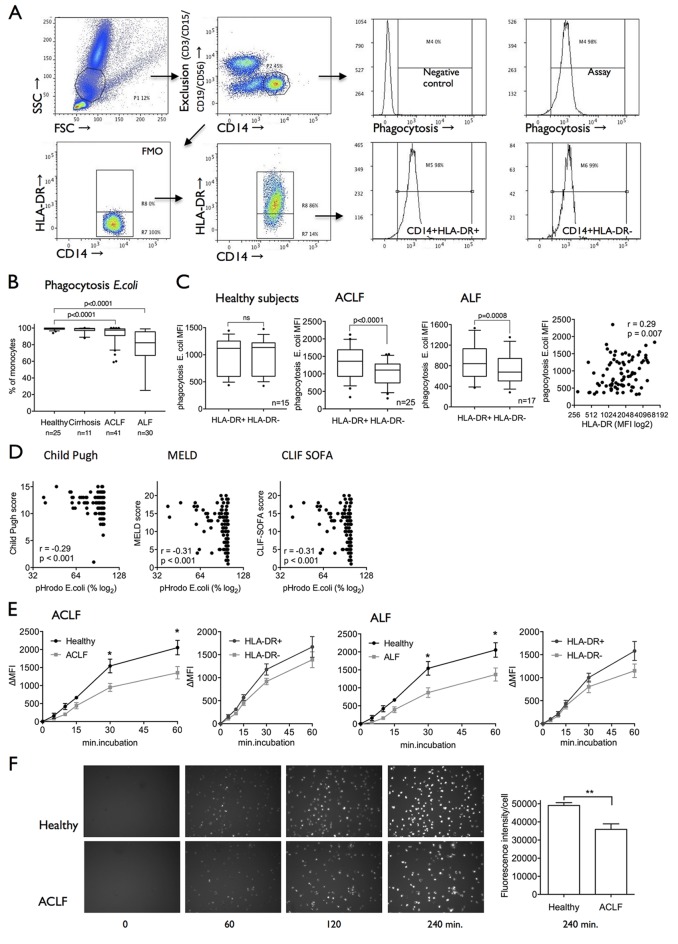
Impaired bacterial uptake and clearance in ACLF is linked to M-MDSC expansion and disease severity. (A) FACS plots showing the methodology of the newly developed whole blood ex vivo phagocytosis assay for different monocyte subsets (CD14^+^HLA-DR^+^ and CD14^+^HLA-DR^−^). Using a lineage exclusion panel (CD3, CD15, CD19, CD56), non-monocytic PBMCs were excluded. (B) In liver failure syndromes (ACLF and ALF), capacity to phagocytose *Escherichia coli* particles was significantly reduced. (C) Ex vivo *E. coli* phagocytosis capacity was reduced in M-MDSC versus CD14+HLA-DR+ monocytes in ACLF and ALF; ex vivo HLA-DR expression correlated with phagocytosis capacity (n=84). (D) Ex vivo phagocytosis capacity correlated with disease severity scores (Child-Pugh, MELD, CLIF-SOFA; n=132). (E) Phagocytosis capacity was reduced and decelerated over time (0–60 min) in CD14+ cells from patients with ACLF and ALF. Pathogen uptake is lower and slower in M-MDSC compared with HLA-DR+ monocytes. Results are shown as ΔMFI, healthy n=9, ACLF n=9, ALF n=9. (F) Cell-IQ-based phagocytosis assay revealed marked and persistent deficiency in the uptake of bacteria in ACLF (0–240 min)(supplementary video). ACLF, acute-on-chronic liver failure; ALF, acute liver failure; CLIF, Consortium on Chronic Liver Failure; FACS, fluorescence-activated cell sorting; FMO, fluorescence minus one control; FSC, forward scatter; MELD, Model for End-Stage Liver Disease; MFI, mean fluorescence intensity; M-MDSC, monocytic myeloid-derived suppressor cells; PBMC, peripheral blood mononuclear cells; SOFA, Sequential Organ Failure Assessment score; SSC, side scatter.

Phagocytosis of bacteria is an important function of myeloid cells with regard to effective clearance of invading microorganisms and during sepsis. The proportion of CD14+ cells able to phagocytose *E. coli* particles was significantly reduced in patients with ACLF (median 97.4%) and ALF (median 82.4%; both p<0.0001, figure 3A,B) when compared with patients with cirrhosis and healthy controls (median 99%). Also, capacity to phagocytose *S. aureus* particles was reduced in both patients with ACLF and ALF and interestingly, the capacity to phagocytose gram-negative (*E. coli*) particles correlated with capacity to phagocytose gram-positive (*S. aureus*) particles (see online supplementary figure 2A). The production of reactive oxidative metabolites following uptake of *E. coli* was preserved in both patients with ACLF and ALF compared with healthy subjects (see online supplementary figure 2B).

10.1136/gutjnl-2017-314184.supp2 Supplementary Figure 2

10.1136/gutjnl-2017-314184.supp8 Supplementary video

A phagocytosis assay was designed to differentially assess phagocytosis capabilities of CD14+HLA-DR+ monocytes compared with CD14^+^HLA-DR^−^ M-MDSC (figure 3A). In patients with ACLF and ALF, M-MDSC revealed significantly reduced phagocytosis of *E. coli* particles ex vivo when compared with HLA-DR+ monocytes. Phagocytosis capacity of *E. coli* positively correlated with the degree of HLA-DR expression on all CD14+ expressing cells (figure 3C) and TNF-α production in response to LPS (r=0.35, p=0.04, n=36). Similar to HLA-DR expression, phagocytosis indices correlated with disease severity scores (Child-Pugh score, MELD, SIRS score, CLIF-SOFA score; figure 3D). Taken together, ex vivo experiments reveal that CD14+ cells, in particular the M-MDSC subset, exhibit a marked and persistent deficiency in the uptake and clearance of bacteria in patients with ALF and ACLF (figure 3E,F and online supplementary figure 3).

10.1136/gutjnl-2017-314184.supp3 Supplementary Figure 3

### Transcriptional analysis of circulating monocytes links impaired phagocytosis to downregulation of TLR pathways

**Figure 4:**
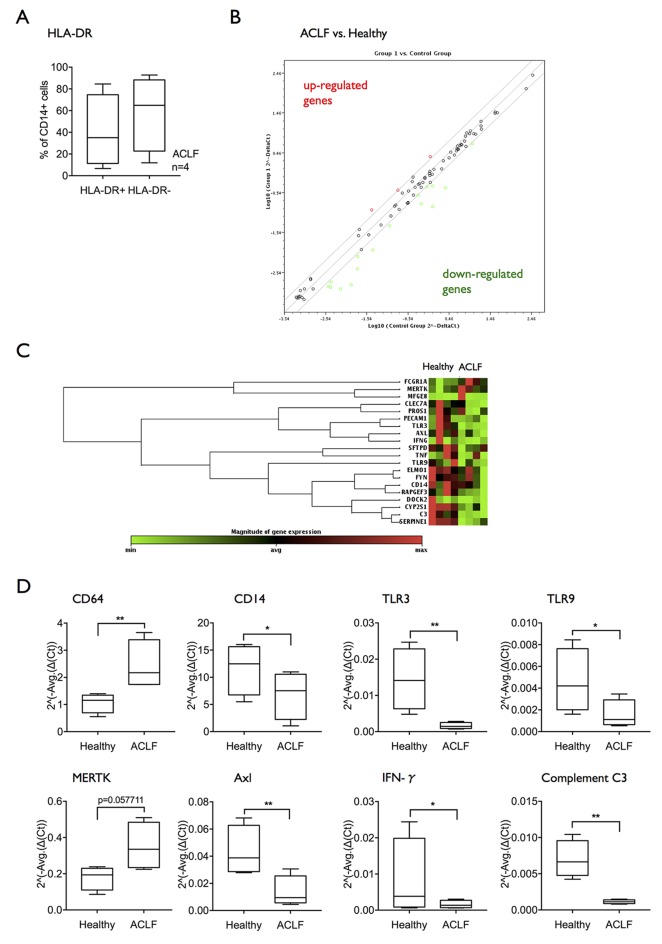
mRNA expression of Toll-like receptors is reduced in CD14+ cells from patients with ACLF. (A) A phagocytosis-specific PCR array was performed using mRNA extracted from isolated CD14+ cells (n=4 healthy subjects; n=4 ACLF). Selected patients with ACLF showed an expansion of the M-MDSC subset. (B) Scatter plot showing genes upregulated or downregulated more than twofold in ACLF compared with healthy subjects. (C) Clustergram showing the differential mRNA expression of genes regulated at least twofold. (D) Differential mRNA expression in selected genes of interest: CD64, CD14, TLR3, TLR9, MERTK, Axl, IFN-γ, complement C3. Data are shown as 2^(-Average(ΔCt)). ACLF, acute-on-chronic liver failure; IFN-γ; interferon-γ; MERTK, Mer Tyrosine Kinase; M-MDSC, monocytic myeloid-derived suppressor cells.

In order dissect which intracellular pathways may be involved in defective bacterial clearance and innate responses in ACLF, a phagocytosis-specific RT-PCR array was performed comparing mRNA extracts from CD14+ cells of patients with ACLF bearing an expanded M-MDSC population compared with healthy subjects (figure 4A). Differential expression more than twofold revealed higher expression of three genes (FcγR1/CD64, MERTK and MFGE8) and reduced expression of 16 genes interestingly including TLR-3 and TLR-9, CD14 and interferon-γ. The findings indicate multiple defective pathways of innate immune responses in CD14+ cells from patients with ACLF with marked reductions in expression of diverse TLRs and in Th1 responses, and simultaneous increases in pathways associated with the clearance of necrotic and apoptotic cells (figure 4B–D, table 2). The striking reduction in expression of transcripts of a number of different TLR pathways led to the hypothesis that reduction in TLR signalling may impair TLR-driven innate immune responses and therefore represent a potential therapeutic target.

**Table 2:** Genes overexpressed (A) and underexpressed (B) in patients with ACLF versus healthy controls. Quantitative RT-PCR data from n=4 patients of each group in triplicate. Fold difference >1.99

A			B		
Gene symbol	Fold regulation	p Value	Gene symbol	Fold regulation	p Value
FCGR1A	2.3014	0.000418	AXL	−3.8622	0.0001
MERTK	1.9998	0.057711	C3	−5.9677	0.007653
MFGE8	2.7807	0.060632	CD14	−2.1498	0.040977
			CLEC7A	−6.1628	0.536001
			CSF1	−2.4128	0.174415
			CYP2S1	−4.0056	0.000001
			DOCK2	−9.0148	0.322467
			ELMO1	−2.0128	0.066372
			FYN	−2.0566	0.074544
			IFNG	−2.6871	0.048288
			PECAM1	−2.7643	0.033099
			RAPGEF3	−2.3415	0.012308
			SERPINE1	−4.7454	0.006937
			SFTPD	−2.4618	0.00293
			TLR3	−8.5662	0.000047
			TLR9	−3.1654	0.041817

### Generation of the immunosuppressive M-MDSC population in patients with ACLF may result from circulating bacterial products stimulating TLR pathways and circulating cytokines

**Figure 5:**
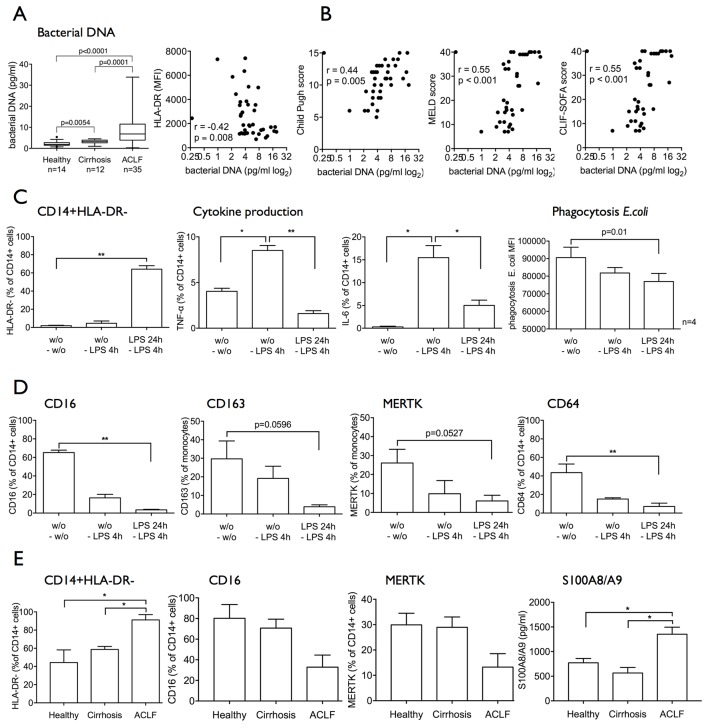
Circulating bacterial products and cytokines in patients with ACLF may lead to the generation of an immunosuppressive M-MDSC-like population. (A) Bacterial DNA levels in whole blood were significantly elevated in patients with ACLF and negatively correlated with HLA-DR expression. (B) Accordingly, bacterial DNA levels in whole blood positively correlated with markers of disease severity (Child-Pugh, MELD, CLIF-SOFA). (C and D) Healthy CD14+ cells were incubated with TLR-4 ligand LPS for 24 hours and subsequent 4 hours (n=4 independent experiments). HLA-DR expression, cytokine responses to LPS, phagocytosis capacity (C) and phenotype (CD16, CD163, MERTK, CD64, TLR-4, TLR-3, TLR-9) (D) were assessed. Recurrent LPS stimulation leads to the generation of an immunosuppressive HLA-DR^low^CD16^low^CD163^low^MERTK^low^ M-MDSC-like population. Data are presented as % of CD14+ cells or MFI, respectively. Paired t-tests. (E) ACLF plasma containing bacterial products and modulated cytokine levels led to generation of an HLA-DR^low^CD16^low^MERTK^low^ M-MDSC-like population. S100A8/A9 protein secretion into the supernatants response to LPS was significantly increased. Plasma from n=3 healthy subjects, n=3 cirrhotics, n=6 ACLF, Mann-Whitney U tests. ACLF, acute-on-chronic liver failure; CLIF, Consortium on Chronic Liver Failure; IL-6, interleukin-6; LPS, lipopolysaccharide; MELD, Model for End-Stage Liver Disease; MERTK, Mer Tyrosine Kinase; MFI, mean fluorescence intensity; M-MDSC, monocytic myeloid-derived suppressor cells; SOFA, Sequential Organ Failure Assessment score; TLR-4, Toll-like receptor 4; TNF-α, tumour necrosis factor-alpha.

In addition to impaired pathogen responses and clearance mechanisms, we also detect elevated levels of bacterial DNA in whole blood from patients with ACLF compared with cirrhosis and in cirrhosis compared with healthy subjects. Furthermore, bacterial DNA titres negatively correlated with the expansion of CD14^+^HLA-DR^−^ M-MDSCs (figure 5A) and with a number of indices of disease severity (figure 5B).

We therefore hypothesised that continual exposure to PAMPs gives rise to the immunosuppressive M-MDSC with impaired antimicrobial responses as described in vivo. In an in vitro model administering recurrent LPS stimulation^25^ as well as recurrent Pam3CSK4 stimulation, we observed (1) expansion of M-MDSC characterised by reduced expression of HLA-DR, CD16, CD163, MERTK, CD64; (2) attenuated proinflammatory responses to LPS; and (3) reduced phagocytosis capacity (figure 5C–E and online supplementary figures 4A–D and 5A–D). Overall the results were more pronounced in the CD14+HLA-DR− MDSC subset (see online supplementary figures 4 and 5).

Simultaneous repeated stimulation with LPS and Pam3CSK4 also results in an expansion of M-MDSC, reduced TLR-evoked proinflammatory responses along with an HLA-DR_low_CD16_low_CD163_low_MERTK_low_CD64_low_ phenotype (see online supplementary figure 6). Recurrent PAMP stimulation reduced the numbers of viable HLA-DR+ monocytes (Annexin V-/7-AAD-), but did not significantly alter the survival of M-MDSC (see online supplementary figures 4E, 5E, 6E).

10.1136/gutjnl-2017-314184.supp6 Supplementary Figure 6

In addition to PAMPs, soluble factors such as cytokines have been reported to induce M-MDSCs. We and others had previously shown that numerous cytokines (TNF-α, IL-6, IL-10, IL-8) are upregulated in ACLF in comparison to cirrhosis and healthy subjects, respectively.^23 27^ We therefore studied the effect of ACLF plasma on healthy CD14+ monocytes in vitro and indeed observed the expansion of the M-MDSC population revealing a CD16_low_MERTK_low_ phenotype. Also, the secretion of S100A8/A9 protein levels significantly increased in the cell culture supernatants of CD14+ cells after incubation with ACLF plasma. Similar to data reported above, the viability of generated M-MDSC was not reduced compared with CD14+HLA-DR+ monocytes. The data implicate the likely involvement of other soluble mediators such as proinflammatory cytokines causing the generation of M-MDSC in patients with ACLF (figure 5E).

### In vitro TLR-3 stimulation reduces the frequency of M-MDSCs and augments antimicrobial responses in ACLF

The downregulated expression of TLRs raised the question whether regulation of innate immune function by TLRs was attenuated, and whether targeted TLR agonists may have the potential to reduce the frequency of M-MDSC. Previous reports and studies in man have identified the therapeutic use of TLR-3 agonism therapy as an adjuvant for vaccinations as well as for induction of antitumour immune responses by decreasing the frequency of MDSC.^28–31^ Moreover, studies demonstrated that TLR-3 agonist polyI:C promoted phagocytosis of bacteria in vitro.^32^

**Figure 6:**
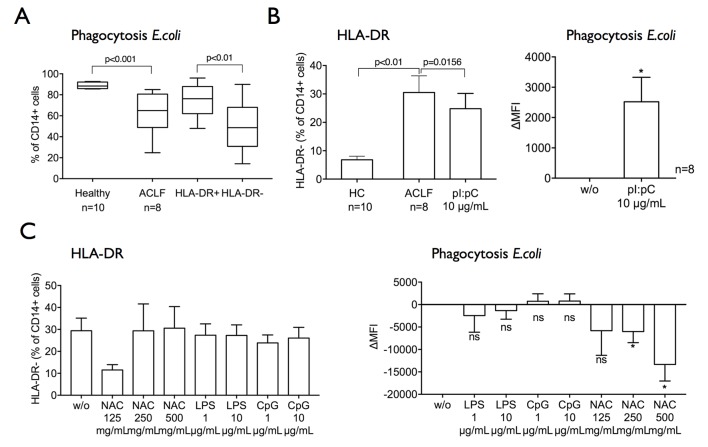
HLA-DR expression and phagocytosis capacity are enhanced by Toll-like receptor 3 agonism. (A) Monocytes cultured in plasma from patients with ACLF compared with healthy plasma (24 hours) develop a defect in phagocytosis capacity, which is more pronounced in the M-MDSC subset. (B) Treatment of CD14+ cells with Toll-like receptor 3 agonist polyI:C (for 6 hours, n=8) significantly reduced the M-MDSC subset and improved phagocytosis capacity. (C) CD14+ cells were treated with LPS, CpG (for 6 hours; n=6) or NAC (for 48 hours, n=7) ex vivo. The CD14+HLA-DR− M-MDSC subset and phagocytosis capacity were assessed. Data are presented as % of CD14+ cells and ΔMFI, in comparison to the untreated sample, respectively; Wilcoxon tests. ACLF, acute-on-chronic liver failure; CpG, CpG oligodeoxynucleotide; HC, healthy controls; LPS, lipopolysaccharide; MFI, mean fluorescence intensity; M-MDSC, monocytic myeloid-derived suppressor cells; NAC, N-acetylcysteine; ns, not significant.

To explore the effect of different TLR agonists on innate immune responses, we incubated healthy CD14+ cells in plasma from patients with ACLF in the presence or absence of TLR agonists (TLR-3, TLR-4, TLR-9). Interestingly, TLR-3 agonist polyI:C significantly reduced the proportions of M-MDSC population while simultaneously enhancing their phagocytosis capacity in ACLF (figure 6A,B). Conversely, treatment with TLR-4 and TLR-9 agonists or NFκ-B inhibitor N-acetylcysteine did not reduce or in fact further increased proportions of M-MDSC and further impaired bacterial phagocytosis (figure 6C).

## Discussion

In this study, we have shown a marked expansion of M-MDSCs^6 8^ that impair antimicrobial responses in patients with ACLF. Their biological relevance is highlighted through their strong association with indices of disease severity and incidence of infectious complications in these patients. In addition to impairing T cell responses, M-MDSCs are typified by impaired (1) secretion of proinflammatory cytokines in response to stimulation with a wide range of TLR ligands and (2) phagocytosis of bacteria. Analyses of these cells reveal a distinct immunophenotype characterised by reduced expression of tissue scavenger, costimulatory and phagocytosis receptors (eg, MERTK, CD64, CD86, CD163).

M-MDSCs are not only elevated in patients where primary infection has been the trigger for the development of ACLF but their persistence is also linked with the development of secondary infections. These data echo data from other inflammatory pathologies where the expansion of these cells^6 8^ correlates with poor prognosis.^9 10 13–19 33–35^ These observations urge to prospectively evaluate the role of M-MDSCs as biomarkers of infection and survival in large cohorts of patients with cirrhosis and ACLF.

Our data also reveal a less pronounced but significant elevation in M-MDSC in patients with cirrhosis. Here, we suggest that they are likely to evolve as a consequence of continual exposure to elevated concentrations of gut-derived PAMPs and proinflammatory mediators that are probably implicated in disease progression. Further studies are required to detail how these cells evolve and their role and migratory patterns in the liver and other tissue sites.

The generation of M-MDSC involves a complex interplay of numerous soluble circulating factors.^36 37^ Using in vitro models, we were able to recapitulate the M-MDSC phenotype with comparable defects of innate immune function, suggesting that the generation and persistence of M-MDSC in ACLF could be related to repetitive stimulation by elevated circulating titres of PAMPs. This hypothesis is further strengthened by our observation of increased bacterial DNA levels in patients with ACLF, which correlated with the frequency of M-MDSCs. Among TLRs, TLR-2/6^38^ and TLR-4^39–41^ ligands have been reported to promote MDSC population. Consistent with these findings, our data reveal that recurrent stimulation of TLR-2 and/or TLR-4 induces a similar suppressive M-MDSC-like population as described ex vivo.

Given that both ALF and ACLF are conditions characterised by a profound activation of SIRS responses, in addition to continual PAMP exposure, we also propose that increases in circulating inflammatory mediators also contribute to the expansion and differentiation of the M-MDSCs. Our in vitro data support this concept where we demonstrate that incubation of CD14+ cells in the presence of ACLF plasma induces striking increases in their numbers. Taken together, our observations indicate the expansion and differentiation of myeloid cells into M-MDSC in patients with ACLF occur as a consequence of perpetual immune stimulation from microbial and non-microbial inflammatory cues.

The transcriptional profile of CD14+ myeloid cells in ACLF suggests these cells are functionally reprogrammed to promote uptake of apoptotic cells while concomitantly suppressing T cell activation, TLR-triggered proinflammatory responses and phagocytosis of bacteria. A similar transcriptional profile has been recently reported in patients with septic shock where the leucocyte transcriptome is reprogrammed towards resolution of tissue injury and with concomitant downregulation of genes that prime innate and adaptive antimicrobial responses.^42^ Further evidence to support this theory is provided in experimental lung injury models where, following clearance of necrotic/apoptotic cells, macrophages have an attenuated uptake of bacteria and augmented secretion of anti-inflammatory mediators.^43^ A similar mechanism may explain how these immunological defects evolve in ACLF, where the host response to severe tissue injury and organ dysfunction is skewed towards tissue repair responses to the detriment of antimicrobial programmes. We therefore hypothesised that selective activation of TLR pathways may represent an immunotherapeutic strategy in order to reverse M-MDSC expansion and improve antimicrobial responses in ACLF.

Deng *et al* had observed that TLR-3 agonist polyI:C promoted bacterial uptake in murine peritoneal macrophages via a TRIF-IRF-3-mediated mechanism.^32^ TLR-3 is classically activated by double-stranded RNA from viruses, but may also be released from degraded bacteria and necrotic cells.^44 45^ In experimental models, TLR-3 activation was associated with an increased innate immune cell infiltrate and enhanced infection clearance.^46 47^ In tumour models, TLR-3 activation reverses the expansion and immunosuppressive function of MDSCs^29 30^ and has been used as an immunotherapeutic agent to promote tumouricidal responses.^29 48 49^ Consistent with these observations, TLR-3 reduced the frequency of M-MDSC and augments innate immune responses and pathogen uptake in patients with ACLF. These findings may relate to the fact that TLR-3 differentially regulates phagocytosis and may ‘rebalance’ the phagocytosis processes which are skewed predominantly towards apoptotic cell clearance in ACLF to the detriment of antimicrobial responses. Given that TLR-3 activation has been shown to be protective in experimental models of chronic liver injury, a TLR-3 agonist may improve antibacterial responses without having a detrimental effect on tissue repair processes.^32 50^ Further work using in vitro and in vivo models of disease is required to further understand how activation of the TLR-3 pathway modulates inflammatory and antimicrobial responses before considering its use in human studies.

Although M-MDSCs correlate with indices of disease severity, incidence of infectious complications, the data presented in this study strongly suggest but cannot conclusively prove that the increased frequencies of M-MDSC are responsible for reduced pathogen clearance in vivo. Moreover, the immune read-outs developed here do not represent point-of care testing that could be introduced for clinical use in multicentre cohort studies. Future prospective work is therefore required to further evaluate the utility of M-MDSC as a predictive biomarker of infection susceptibility and an immunotherapeutic target.

In conclusion, we describe a marked expansion of M-MDSC in ACLF. These cells are characterised by attenuated immune responses to pathogens, defective pathogen clearance mechanisms and a higher incidence of overt infectious complications. Perpetual exposure to elevated concentrations of PAMPs and inflammatory cytokines involved in SIRS response play a pathogenic role in the expansion of this immunosuppressive population. In vitro data indicate that TLR-3 agonism may reduce the frequency of M-MDSC and therefore merit further evaluation as potential novel immunotherapeutic strategy to restore antimicrobial responses and reduce infectious complications in patients with ACLF.

10.1136/gutjnl-2017-314184.supp4 Supplementary Figure 4

10.1136/gutjnl-2017-314184.supp5 Supplementary Figure 5

## References

[1] Moreau R, Jalan R, Gines P, et al Acute-on-chronic liver failure is a distinct syndrome that develops in patients with acute decompensation of cirrhosis. Gastroenterology 2013;144 37:1426 1437–1437.e9. 10.1053/j.gastro.2013.02.042 23474284

[2] Bonnel AR, Bunchorntavakul C, Reddy KR Immune dysfunction and infections in patients with cirrhosis. Clin Gastroenterol Hepatol 2011;9:727–38. 10.1016/j.cgh.2011.02.031 21397731

[3] Albillos A, Lario M, Álvarez-Mon M Cirrhosis-associated immune dysfunction: distinctive features and clinical relevance. J Hepatol 2014;61:1385–96. 10.1016/j.jhep.2014.08.010 25135860

[4] Bajaj JS, O’Leary JG, Reddy KR, et al Second infections independently increase mortality in hospitalized patients with cirrhosis: the north american consortium for the study of end-stage liver disease (NACSELD) experience. Hepatology 2012;56:2328–35. 10.1002/hep.25947 22806618 PMC3492528

[5] Arvaniti V, D’Amico G, Fede G, et al Infections in patients with cirrhosis increase mortality four-fold and should be used in determining prognosis. Gastroenterology 2010;139:1246–56. 10.1053/j.gastro.2010.06.019 20558165

[6] Peranzoni E, Zilio S, Marigo I, et al Myeloid-derived suppressor cell heterogeneity and subset definition. Curr Opin Immunol 2010;22:238–44. 10.1016/j.coi.2010.01.021 20171075

[7] Marvel D, Gabrilovich DI Myeloid-derived suppressor cells in the tumor microenvironment: expect the unexpected. J Clin Invest 2015;125:3356–64. 10.1172/JCI80005 26168215 PMC4588239

[8] Bronte V, Brandau S, Chen SH, et al Recommendations for myeloid-derived suppressor cell nomenclature and characterization standards. Nat Commun 2016;7:12150 10.1038/ncomms12150 27381735 PMC4935811

[9] Hoechst B, Ormandy LA, Ballmaier M, et al A new population of myeloid-derived suppressor cells in hepatocellular carcinoma patients induces CD4(+)CD25(+)Foxp3(+) T cells. Gastroenterology 2008;135:234–43. 10.1053/j.gastro.2008.03.020 18485901

[10] Arihara F, Mizukoshi E, Kitahara M, et al Increase in CD14+HLA-DR -/low myeloid-derived suppressor cells in hepatocellular carcinoma patients and its impact on prognosis. Cancer Immunol Immunother 2013;62:1421–30. 10.1007/s00262-013-1447-1 23764929 PMC11029267

[11] Hammerich L, Tacke F Emerging roles of myeloid derived suppressor cells in hepatic inflammation and fibrosis. World J Gastrointest Pathophysiol 2015;6:43–50. 10.4291/wjgp.v6.i3.43 26301117 PMC4540705

[12] Sander LE, Sackett SD, Dierssen U, et al Hepatic acute-phase proteins control innate immune responses during infection by promoting myeloid-derived suppressor cell function. J Exp Med 2010;207:1453–64. 10.1084/jem.20091474 20530204 PMC2901069

[13] Janols H, Bergenfelz C, Allaoui R, et al A high frequency of MDSCs in Sepsis patients, with the granulocytic subtype dominating in gram-positive cases. J Leukoc Biol 2014;96:685–93. 10.1189/jlb.5HI0214-074R 24929004

[14] Mougiakakos D, Jitschin R, von Bahr L, et al Immunosuppressive CD14+HLA-DRlow/neg IDO+ myeloid cells in patients following allogeneic hematopoietic stem cell transplantation. Leukemia 2013;27:377–88. 10.1038/leu.2012.215 22828446

[15] Yin J, Wang C, Huang M, et al Circulating CD14(+) HLA-DR(-/low) myeloid-derived suppressor cells in leukemia patients with allogeneic hematopoietic stem cell transplantation: novel clinical potential strategies for the prevention and cellular therapy of graft-versus-host disease. Cancer Med 2016;5:1654–69. 10.1002/cam4.688 27109254 PMC4944894

[16] Filipazzi P, Valenti R, Huber V, et al Identification of a new subset of myeloid suppressor cells in peripheral blood of melanoma patients with modulation by a granulocyte-macrophage colony-stimulation factor-based antitumor vaccine. J Clin Oncol 2007;25:2546–53. 10.1200/JCO.2006.08.5829 17577033

[17] Vuk-Pavlović S, Bulur PA, Lin Y, et al Immunosuppressive CD14+HLA-DRlow/- monocytes in prostate Cancer. Prostate 2010;70:443–55. 10.1002/pros.21078 19902470 PMC2935631

[18] Wasmuth HE, Kunz D, Yagmur E, et al Patients with acute on chronic liver failure display "sepsis-like" immune paralysis. J Hepatol 2005;42:195–201. 10.1016/j.jhep.2004.10.019 15664244

[19] Antoniades CG, Berry PA, Davies ET, et al Reduced monocyte HLA-DR expression: a novel biomarker of disease severity and outcome in acetaminophen-induced acute liver failure. Hepatology 2006;44:34–43. 10.1002/hep.21240 16799971

[20] Berres ML, Schnyder B, Yagmur E, et al Longitudinal monocyte human leukocyte antigen-DR expression is a prognostic marker in critically ill patients with decompensated liver cirrhosis. Liver Int 2009;29:536–43. 10.1111/j.1478-3231.2008.01870.x 18795898

[21] Berry PA, Antoniades CG, Carey I, et al Severity of the compensatory anti-inflammatory response determined by monocyte HLA-DR expression may assist outcome prediction in cirrhosis. Intensive Care Med 2011;37:453–60. 10.1007/s00134-010-2099-7 21161643

[22] Bajaj JS, O’Leary JG, Reddy KR, et al Survival in infection-related acute-on-chronic liver failure is defined by extrahepatic organ failures. Hepatology 2014;60:250–6. 10.1002/hep.27077 24677131 PMC4077926

[23] Bernsmeier C, Pop OT, Singanayagam A, et al Patients with acute-on-chronic liver failure have increased numbers of regulatory immune cells expressing the receptor tyrosine kinase MERTK. Gastroenterology 2015;148:603–15. 10.1053/j.gastro.2014.11.045 25479139

[24] Vergis N, Atkinson SR, Knapp S, et al Patients with severe alcoholic Hepatitis given Prednisolone therapy who have high circulating levels of bacterial DNA are at increased risk for developing infections. Gastroenterology (Published Online First: 30 December 2016).

[25] Pena OM, Pistolic J, Raj D, et al Endotoxin tolerance represents a distinctive state of alternative polarization (M2) in human mononuclear cells. J Immunol 2011;186:7243–54. 10.4049/jimmunol.1001952 21576504

[26] Sinha P, Okoro C, Foell D, et al Proinflammatory S100 proteins regulate the accumulation of myeloid-derived suppressor cells. J Immunol 2008;181:4666–75. 10.4049/jimmunol.181.7.4666 18802069 PMC2810501

[27] Clària J, Stauber RE, Coenraad MJ, et al Systemic inflammation in decompensated cirrhosis: characterization and role in acute-on-chronic liver failure. Hepatology 2016;64:1249–64. 10.1002/hep.28740 27483394

[28] Nicodemus CF, Wang L, Lucas J, et al Toll-like receptor-3 as a target to enhance bioactivity of Cancer immunotherapy. Am J Obstet Gynecol 2010;202 8:608.e1–608.e8. 10.1016/j.ajog.2009.12.001 20080226

[29] Ho V, Lim TS, Lee J, et al TLR3 agonist and Sorafenib combinatorial therapy promotes immune activation and controls hepatocellular carcinoma progression. Oncotarget 2015;6:27252–66. 10.18632/oncotarget.4583 26287667 PMC4694987

[30] Le Noci V, Sommariva M, Tortoreto M, et al Reprogramming the lung microenvironment by inhaled immunotherapy fosters immune destruction of tumor. Oncoimmunology 2016;5:e1234571 10.1080/2162402X.2016.1234571 27999750 PMC5139640

[31] Cardinaud S, Urrutia A, Rouers A, et al Triggering of TLR-3, -4, NOD2, and DC-SIGN reduces viral replication and increases T-cell activation capacity of HIV-infected human dendritic cells. Eur J Immunol 2017;47:818–29. 10.1002/eji.201646603 28266028

[32] Deng T, Feng X, Liu P, et al Toll-like receptor 3 activation differentially regulates phagocytosis of bacteria and apoptotic neutrophils by mouse peritoneal macrophages. Immunol Cell Biol 2013;91:52–9. 10.1038/icb.2012.45 22986631

[33] Cheron A, Floccard B, Allaouchiche B, et al Lack of recovery in monocyte human leukocyte antigen-DR expression is independently associated with the development of sepsis after major trauma. Crit Care 2010;14:R208 10.1186/cc9331 21092108 PMC3220028

[34] Lin Y, Gustafson MP, Bulur PA, et al Immunosuppressive CD14+HLA-DR(low)/- monocytes in B-cell non-Hodgkin lymphoma. Blood 2011;117:872–81. 10.1182/blood-2010-05-283820 21063024 PMC3035079

[35] Laborde RR, Lin Y, Gustafson MP, et al Cancer vaccines in the world of immune suppressive monocytes (CD14(+)HLA-DR(lo/neg) Cells): The Gateway to Improved responses. Front Immunol 2014;5:147 10.3389/fimmu.2014.00147 24772111 PMC3983500

[36] Lechner MG, Liebertz DJ, Epstein AL Characterization of cytokine-induced myeloid-derived suppressor cells from normal human peripheral blood mononuclear cells. J Immunol 2010;185:2273–84. 10.4049/jimmunol.1000901 20644162 PMC2923483

[37] Lechner MG, Megiel C, Russell SM, et al Functional characterization of human Cd33+ and Cd11b+ myeloid-derived suppressor cell subsets induced from peripheral blood mononuclear cells co-cultured with a diverse set of human tumor cell lines. J Transl Med 2011;9:90 10.1186/1479-5876-9-90 21658270 PMC3128058

[38] Maruyama A, Shime H, Takeda Y, et al Pam2 lipopeptides systemically increase myeloid-derived suppressor cells through TLR2 signaling. Biochem Biophys Res Commun 2015;457:445–50. 10.1016/j.bbrc.2015.01.011 25596131

[39] Bunt SK, Clements VK, Hanson EM, et al Inflammation enhances myeloid-derived suppressor cell cross-talk by signaling through Toll-like receptor 4. J Leukoc Biol 2009;85:996–1004. 10.1189/jlb.0708446 19261929 PMC2698586

[40] Van Rompaey N, Le Moine A Myeloid-derived suppressor cells: characterization and expansion in models of endotoxemia and transplantation. Methods Mol Biol 2011;677:169–80. 10.1007/978-1-60761-869-0_12 20941610

[41] Fontaine M, Planel S, Peronnet E, et al S100A8/A9 mRNA induction in an ex vivo model of endotoxin tolerance: roles of IL-10 and IFNγ. PLoS One 2014;9:e100909 10.1371/journal.pone.0100909 24956170 PMC4067416

[42] Cazalis MA, Lepape A, Venet F, et al Early and dynamic changes in gene expression in septic shock patients: a genome-wide approach. Intensive Care Med Exp 2014;2:20 10.1186/s40635-014-0020-3 26215705 PMC4512996

[43] Medeiros AI, Serezani CH, Lee SP, et al Efferocytosis impairs pulmonary macrophage and lung antibacterial function via PGE2/EP2 signaling. J Exp Med 2009;206:61–8. 10.1084/jem.20082058 19124657 PMC2626688

[44] Yu L, Wang L, Chen S Endogenous toll-like receptor ligands and their biological significance. J Cell Mol Med 2010;14:2592–603. 10.1111/j.1582-4934.2010.01127.x 20629986 PMC4373479

[45] Sotolongo J, España C, Echeverry A, et al Host innate recognition of an intestinal bacterial pathogen induces TRIF-dependent protective immunity. J Exp Med 2011;208:2705–16. 10.1084/jem.20110547 22124111 PMC3244044

[46] Wu J, Meng Z, Jiang M, et al Toll-like receptor-induced innate immune responses in non-parenchymal liver cells are cell type-specific. Immunology 2010;129:363–74. 10.1111/j.1365-2567.2009.03179.x 19922426 PMC2826681

[47] Davis CG, Chang K, Osborne D, et al TLR3 agonist improves survival to secondary pneumonia in a double injury model. J Surg Res 2013;182:270–6. 10.1016/j.jss.2012.09.039 23083640

[48] Adams S Toll-like receptor agonists in cancer therapy. Immunotherapy 2009;1:949–64. 10.2217/imt.09.70 20563267 PMC2886992

[49] Chew V, Tow C, Huang C, et al Toll-like receptor 3 expressing tumor parenchyma and infiltrating natural killer cells in hepatocellular carcinoma patients. J Natl Cancer Inst 2012;104:1796–807. 10.1093/jnci/djs436 23197495 PMC3814220

[50] Liu C, Zhang C, Lu H, et al Poly(I:c) induce bone marrow precursor cells into myeloid-derived suppressor cells. Mol Cell Biochem 2011;358:317–23. 10.1007/s11010-011-0982-3 21744070

