# Supplementary material for: CD14+ CD15− HLA-DR− myeloid-derived suppressor cells impair antimicrobial responses in patients with acute-on-chronic liver failure
Source: Gut. 2017 Jun 7;67(6):1155–67. doi: 10.1136/gutjnl-2017-314184 (PMC5969362; doi:10.1136/gutjnl-2017-314184)
Supplement: Supplementary Methods [file gutjnl-2017-314184supp007.pdf]

Supplementary Methods

Whole Blood Phagocytosis assay

The pHrodo^{TM} E. coli Red BioParticles® Phagocytosis Kit for Flow Cytometry was purchased from Invitrogen, Paisley, UK. The particles are inactivated, unopsonized *Escherichia coli* (K-12 strain), highly sensitive, fluorogenic particles for the detection of phagocytic ingestion. The unique pHrodo^{TM} dye-based system measures phagocytic activity based on acidification of particles as they are ingested, hereby eliminating the detection of bound but not internalized particles. The optimal absorption and fluorescence emission maxima are approximately 560 nm and 585 nm, respectively.

The instructions of the manufacturer were precisely followed. Heparinated whole blood was collected and stored on ice for no longer than two hours prior to the phagocytosis assay. 20 μL of E. coli Red BioParticles® were added to 100 μL of whole blood in sterile 5ml conical tubes (1:10 pathogen to whole blood ratio). As a negative control, 20 μL of reconstitution buffer were added to 100 μL whole blood. The tubes were incubated at 37°C for 15 minutes, and placed on ice to stop the reaction in precise time. 100 µL of lysis buffer were added for 5 min. at room temperature (RT), followed by addition of 1mL of reconstitution buffer for 5 min. at RT. Samples were centrifuged at 350g for 5 min. at RT, and the supernatant was removed. After a wash step, the cells were stained with antibodies against CD14 (APC-H7), CD16 (PercP-Cy5.5), HLA-DR (APC) and CD3, CD15, CD19, CD56, all bound to FITC in order to exclude PBMC other than monocytes, for 20 min. at 4°C. After another wash step, cells were acquired on the BD FACS CANTO II flow cytometer. Monocytes and their specific sub-populations were gated to assess bioparticle internalization as shown in Figure 3A. For the phagocytosis time course experiments (Figure 3E) bioparticles were incubated for 0, 5, 10, 15, 30 and 60 minutes in n=9 subjects per group.

The identical protocol was performed also using also pHrodo^{TM} Green S. aureus BioParticles® conjugate. The optimal absorption and fluorescence emission maxima are approximately 509 nm and 533 nm, respectively.

Ex vivo Phagocytosis assay in PBMC

For the assessment of phagocytosis of PBMCs *in vitro*, 500'000 healthy PBMCs per well were cultured (24h) in a 24-well plate in 500µl complete medium (RPMI with 10% FBS plus 1% antibiotics) containing (20%) HC or AoCLF plasma, in a 37°C, 5% CO_2 environment. Cells were stimulated with various agents (polyI:C 10µg/mL, LPS 1 and 10μg/mL, CpG ODN2006 1 and 10µg/mL) for 4h. Cells were then harvested by pipetting vigorously up and down and transferred to sterile flow cytometry tubes. 225 μl of the cell suspension were supplemented by 25 μl of autologous plasma (10%). 20 μl of pHrodo™ E. coli Red BioParticles® were added, and compared to 20 μl complete medium (negative control). Cells were incubated for 60min at 37°C, 5% CO_2 and placed on ice afterwards to stop the reaction in precise time. After a wash step, cells were stained and acquired on the flow cytometer as detailed above.

Phagocytosis of CD14+ cells: Cell IQ

Frozen peripheral blood mononuclear cells from a healthy patient were thawed, washed in RPMI and centrifuged to obtain a cell pellet. CD14+ cells were isolated using Miltenyi CD14 magnetic beads as per the manufacturers' protocol. In each of four wells of a 24-well plate, 500,000 CD14+ cells in complete media were incubated with 25% plasma from two healthy subjects and two patients with ACLF, giving a total volume of 500µl per well. An additional well contained cells in complete media alone to be used as a control. The 24-well plate was incubated for 24 hours at 37°C in 5% CO_2. Purity of the CD14+ cells was confirmed as > 90% by flow cytometry.

After 24 hours, conditioned CD14+ cells were collected from the wells, washed with PBS and resuspended in a new 24-well plate in complete media supplemented with 10% human AB serum (to provide opsonins) to a volume of 500µl per well. 10µl of reconstituted *Escherichia coli* Green BioParticles™ (Invitrogen) were added to each well, except the control well in which 10µl PBS was added.

Real-time cell imaging of the uptake of BioParticles was captured by a Cell-IQ system (CMTechnologies), running Imagen software v2.8.12.0 and analyser version 3.3.0. The 24-well plate was sealed with a Cell-IQ lid to facilitate gas exchange, and then transferred to the Cell-IQ for live cell imaging. Both phase and fluorescence images (469 nm excitation, 35nm bandwith; 525 nm emission, 39nm bandwidth)

were captured over the duration of the experiment, reflecting the intensity of pH-dependent fluorescent emission following BioParticle phagocytosis. Fluorescence intensity was calculated using instrument software.

Quantification of bacterial 16S rDNA levels in whole blood using TaqMan qRT-PCR

The procedure was carried out under strict aseptic conditions as previously reported[24]. Briefly, a 200*µ*l EDTA blood sample was taken. Blood was lysed with 20*µ*l lysozyme/glycogen (20:1) solution (Thermo Fisher, Paisley, UK) for 30 min. at 37°C and subsequently with 20*µ*l of Proteinase K for 60 min. at 56°C (QIAamp QIAamp DNA Blood Mini Kit, Quiagen, Manchester, United Kingdom). DNA was extracted precisely following the manufacturers protocol. Quantitative RT-PCR was performed using TaqMan probes (Applied Biosystems, Thermo Fisher) following a protocol adapted from Jordan et Durso as previously described [24,25]. Primers against the V7-V9 variable region of the 16S gene: Forward RW01: 5'-AACTGGAGGAAGGTGGGGAT-3'. Reverse DG74: 5'-AGGAGGTGATCCAACCGCA-3'. TaqMan Probe: RDR245: 5'-TACAAGGCCCGGGAACGTATTCACCG-3'. The amplification was done using TaqMan Gene Expression Master Mix (Applied Biosciences, Foster City, USA). The protocol included an initial hot start activation for 2 min. at 50°C and then 10 min. at 95°C, following 40 cycles reaction (15 sec. at 95°C, 30 sec. at 60°C, 60 sec. at 72°C) on StepOne Plus PCR machine (Applied Biosciences). As a positive control, *E.coli* ATCC 25922 (American Type Culture Collection, LGC Standards, Teddington, Middlesex, UK) was used. Serial dilutions of *E.coli* DNA were run to generate a standard curve. Bacterial 16S rDNA concentrations were quantified and expressed in pg/ml.

Phagocytosis PCR array

The RT^2 Profiler phagocytosis PCR array (Cat No 330231 PAHS-173ZA) was purchased from SABiosciences, Quiagen, Manchester, United Kingdom. The process from RNA extraction to cDNA synthesis and quantitative RT-PCR was performed exactly as per manufacturer's protocol. RNA was extracted as recommended using RNeasy Mini Kit (Quiagen) from isolated monocytes from n=4

patients with ACLF and defective phagocytosis capacity (pHrodo E.coli <90% of monocytes) and n=4 healthy subjects with normal phagocytosis capacity. cDNA was synthesised using the RT2 First Strand Kit (Quiagen) and quantitative RT PCR was done using the RT^2 SYBR green technology. The array was run on a Life Technologies® ViiA-7® (ViiA 7 Software v1.2) instrument. Analysis of the data was done using the SABiosciences, Quiagen online software.

Supplementary Figure 1. Inflammatory response to Toll-like receptor stimulation is reduced in ACLF compared to healthy subjects.

A. TNF-α- and B. IL-6 production in CD14+ cells upon treatment with different toll-like receptor (TLR) ligands (Pam3CSK4 – ligand to TLR2, Lipopolysaccharide – ligand to TLR4, CpG – ligand to TLR9) were significantly reduced in ACLF. Healthy n=9; ACLF n=8 (Pam3CSK), n=6 (LPS), n=7 (CpG). Mann-Whitney tests.

Supplementary Figure 2. Capacity to phagocytose grampositive bacteria is impaired and related to defective capacity to phagocytose gramnegative bacteria.

A. Capacity to phagocytosis *S.aureus* particles positively correlated with capacity to phagocytose *E.coli* particles. Spearman correlation, n=123. B. Bacterial killing capacity (reactive oxygen metabolite production in response to E. coli uptake) was not significantly different neither in ACLF nor ALF.

Supplementary Figure 3. Impaired bacterial uptake and clearance in ACLF.

A. Cell-IQ based phagocytosis assay. Representation of the number of cells studied. B. Cell-IQ based phagocytosis assay revealed marked and persistent deficiency in the uptake of bacteria in ACLF (video showing phagocytosis uptake in ACLF from 0 to 240 min.).

Supplementary Figure 4. Recurrent lipopolysaccharide treatment in vitro leads to the expansion of an immunesuppressive CD14+HLA-DR- M-MDSC like population. Healthy monocytes were incubated with TLR-4 ligand LPS for 24h and re-exposed to LPS for another 4h (n=4 independent experiments). A. The percentage of CD14+HLA-DR- cells increased following recurrent LPS treatment. B. Cytokine responses to LPS were assessed in CD14+HLA-DR+ and CD14+HLA-DR- cells. C. Phagocytosis capacity was decreased in CD14+HLA-DR+ and CD14+HLA-DR- cells. D. Expression of phenotypic markers (CD16, CD163, MERTK, CD64, TLR-4, TLR-3, TLR-9) was assessed comparing CD14+HLA-DR+ and CD14+HLA-DR- cells, showing higher reduction in expression of these markers in CD14+HLA-DR- cells. E. Viable (AnnexinV-/7-AAD-CD14+) cells were assessed. There is a

decline of viable cells following recurrent LPS treatment, however CD14+HLA-DR- cells are significantly more viable compared to CD14+HLA-DR+. Data is presented as % of monocytes or MFI, respectively. Paired t-tests, ** p>0.01, * p<0.05.

Supplementary Figure 5. Recurrent treatment with lipopetides leads to the generation of a CD14+HLA-DR- M-MDSC like phenotype and function.
Healthy monocytes were incubated with the lipopetide TLR-2 ligand Pam3CSK4 for 24h and re-exposed to Pam3CSK4 for another 4h (n=3 independent experiments). A. The percentage of CD14+HLA-DR- cells significantly increased following recurrent lipopeptide treatment. B. Phagocytosis capacity was assessed in CD14+, CD14+HLA-DR+ and CD14+HLA-DR- cells. A reduction in phagocytosis capacity following recurrent lipopetide treatment was observed, it was significantly more pronounced in CD14+HLA-DR- cells. C. Cytokine responses to LPS were assessed in CD14+ cells and compared between CD14+HLA-DR+ and CD14+HLA-DR- subsets. Cytokine response is significantly reduced after recurrent Pam3-CSK4 treatment. Reduction is more pronounced in CD14+HLA-DR- cells. D. Expression of phenotypic markers (CD16, CD163, MERTK, CD64, TLR-4, TLR-3, TLR-9) was assessed in CD14+ cells and comparing CD14+HLA-DR+ and CD14+HLA-DR- subsets. Expression of CD16, CD163 and CD64 is significantly decreased after recurrent treatment, and more pronounced in CD14+HLA-DR- cells. E. The percentage of viable monocytes (CD14+CD15-AnnexinV-7-AAD-) was assessed and compared between CD14+HLA-DR+ and CD14+HLA-DR- subsets. Viable monocytes decreased after recurrent Pam3CSK4 stimulation, however to a significantly lower extend in CD14+-HLA-DR- M-MDSC like cells. Data is presented as % of monocytes or MFI, respectively. Paired t-tests, ** p>0.01, * p<0.05.

Supplementary Figure 6. Concurrent repetitive treatment with lipopolysaccharides and lipopetides leads to the generation of a M-MDSC like phenotype and function.
Healthy monocytes were concurrently incubated with TLR-4 ligand lipopolysaccharide (LPS) and the lipopetide TLR-2 ligand Pam3CSK4 for 24h and re-exposed to LPS/Pam3CSK4 for another 4h (n=6 independent experiments). A. The

percentage of CD14+HLA-DR- cells following recurrent LPS/Pam3CSK4 treatment increased. B. Phagocytosis capacity was assessed in CD14+, CD14+HLA-DR+ and CD14+HLA-DR- cells. A reduction in phagocytosis capacity following recurrent LPS/Pam3CSK4 treatment was not observed, however phagocytosis capacity is significantly lower in CD14+HLA-DR- cells. C. Cytokine responses to LPS were assessed in CD14+ cells and compared between CD14+HLA-DR+ and CD14+HLA-DR- subsets. TNF-α response is significantly reduced after recurrent LPS/Pam3CSK4 treatment. Reduction is more pronounced in CD14+HLA-DR- cells. D. Expression of phenotypic markers (CD16, CD163, MERTK, CD64, TLR-4, TLR-3, TLR-9) was assessed in CD14+ cells and comparing CD14+HLA-DR+ and CD14+HLA-DR- subsets. Expression of CD16, CD163 and CD64 is significantly decreased after recurrent treatment, and more pronounced in CD14+HLA-DR- cells. E. The percentage of viable monocytes (CD14+CD15-AnnexinV-7-AAD-) was assessed and compared between CD14+HLA-DR+ and CD14+HLA-DR- subsets. Viable monocytes decreased after recurrent LPS/Pam3CSK4 stimulation, however to a lower extend in CD14+-HLA-DR- M-MDSC like cells. Data is presented as % of monocytes or MFI, respectively. Paired t-tests, ** $p>0.01$, * $p<0.05$.
