# Supplementary figures and images for: CD14+ CD15− HLA-DR− myeloid-derived suppressor cells impair antimicrobial responses in patients with acute-on-chronic liver failure
Source: Gut. 2017 Jun 7;67(6):1155–67. doi: 10.1136/gutjnl-2017-314184 (PMC5969362; doi:10.1136/gutjnl-2017-314184)

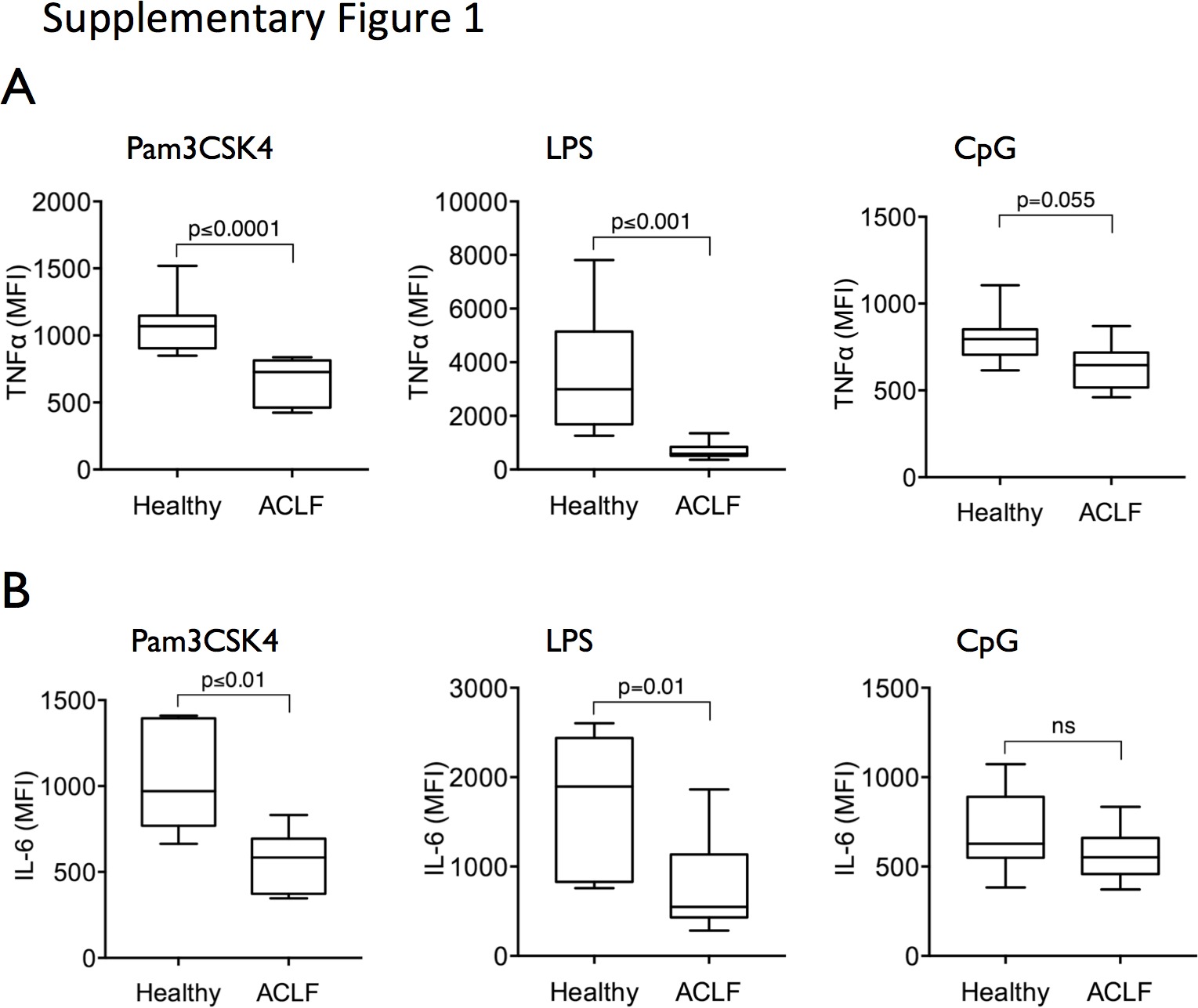

Supplement: Supplementary Figure 1 [file gutjnl-2017-314184supp001.jpg]

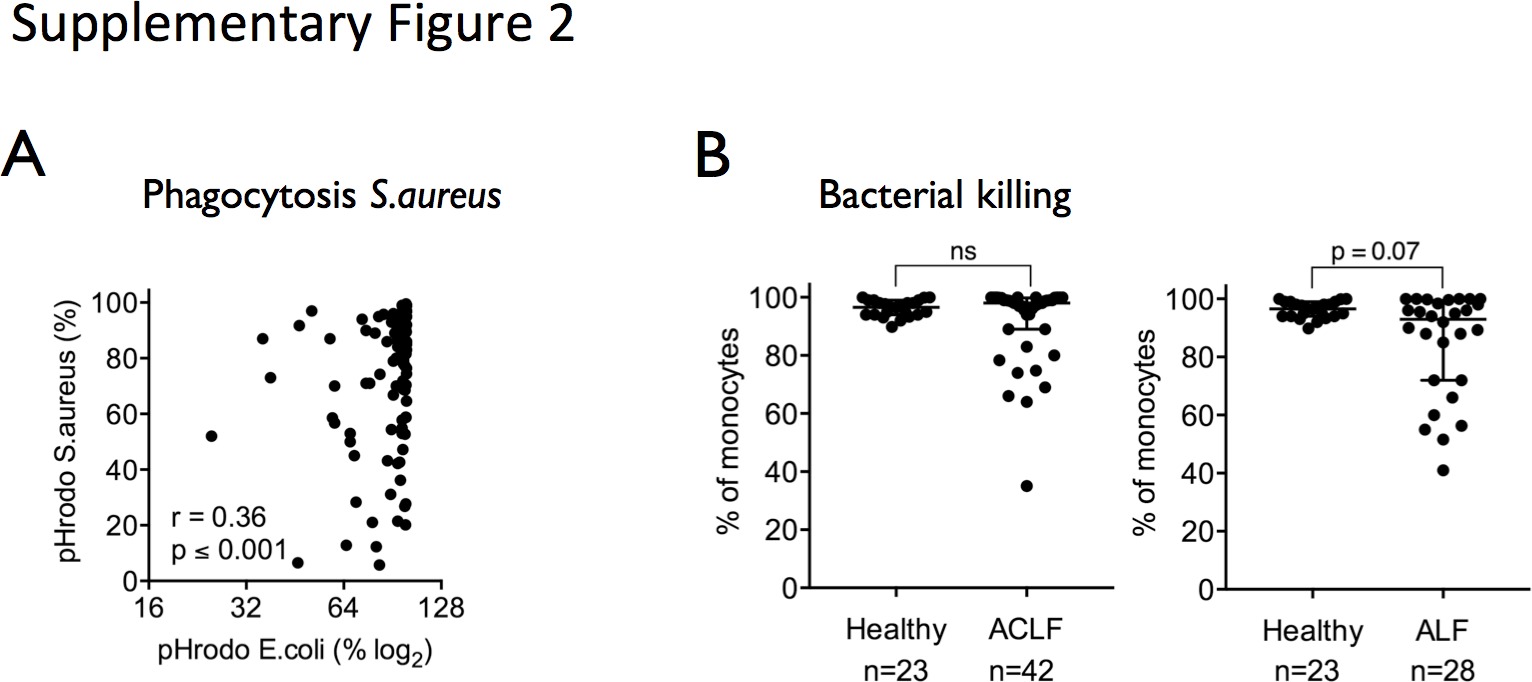

Supplement: Supplementary Figure 2 [file gutjnl-2017-314184supp002.jpg]

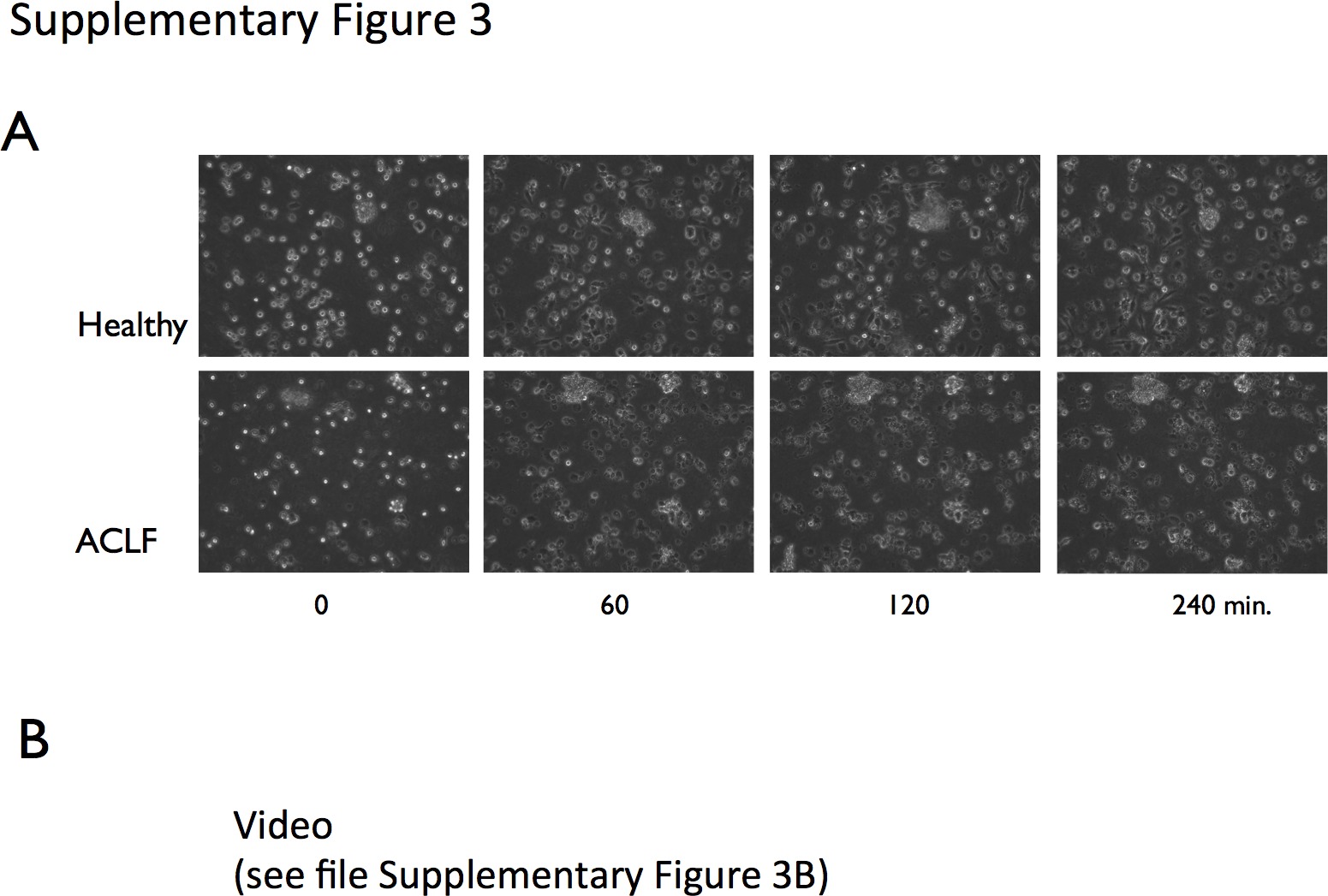

Supplement: Supplementary Figure 3 [file gutjnl-2017-314184supp003.jpg]

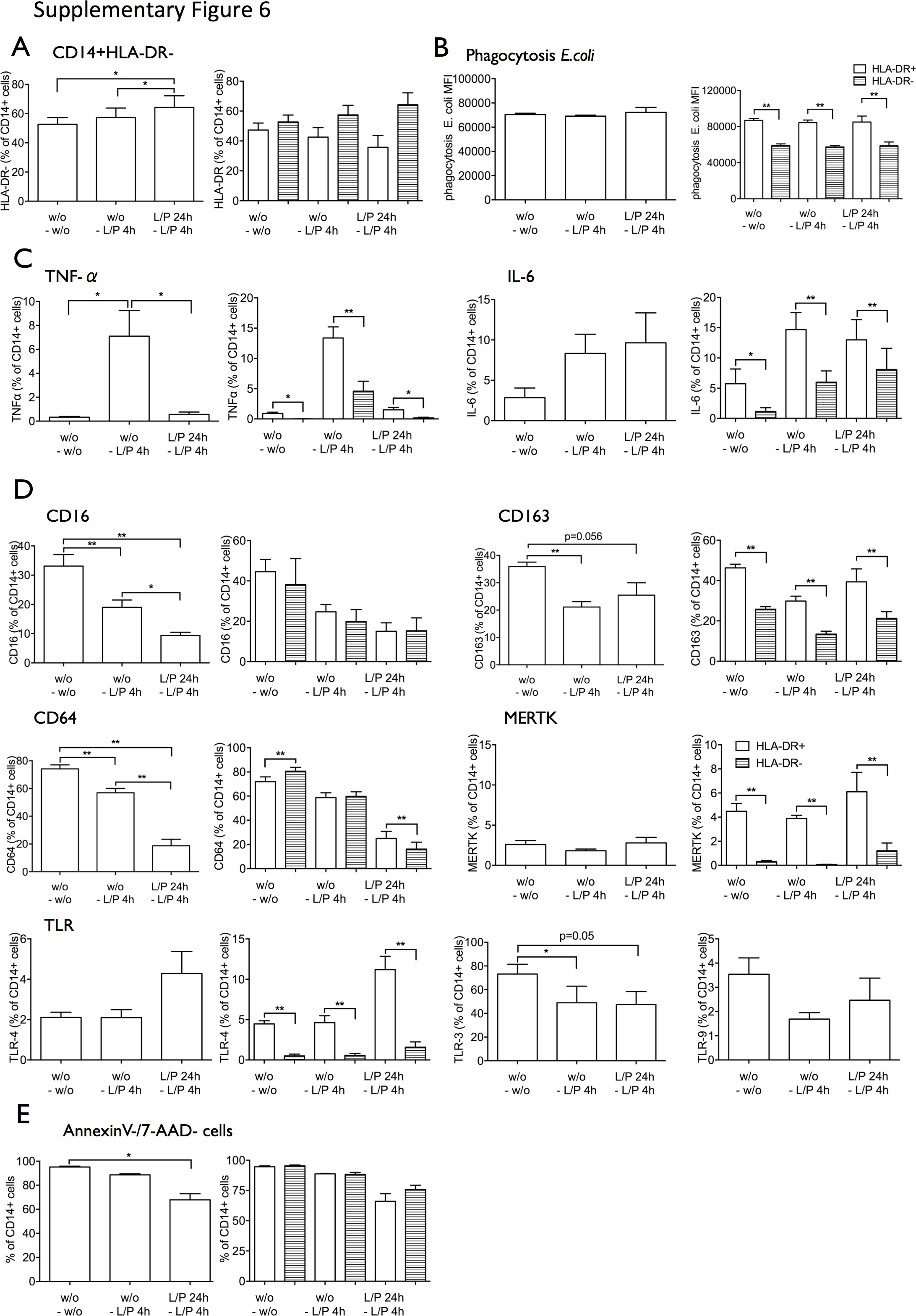

Supplement: Supplementary Figure 6 [file gutjnl-2017-314184supp006.jpg]

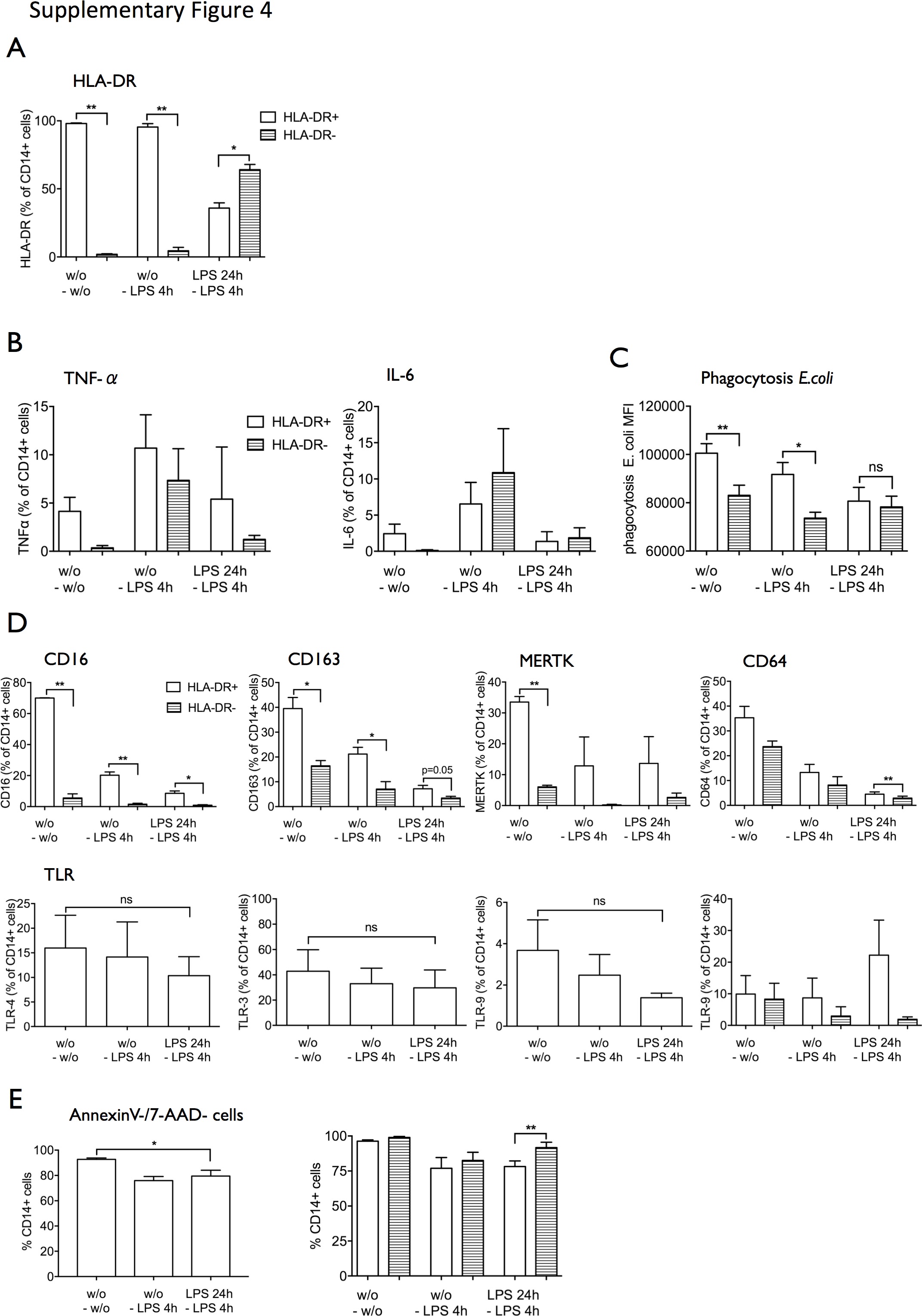

Supplement: Supplementary Figure 4 [file gutjnl-2017-314184supp004.jpg]

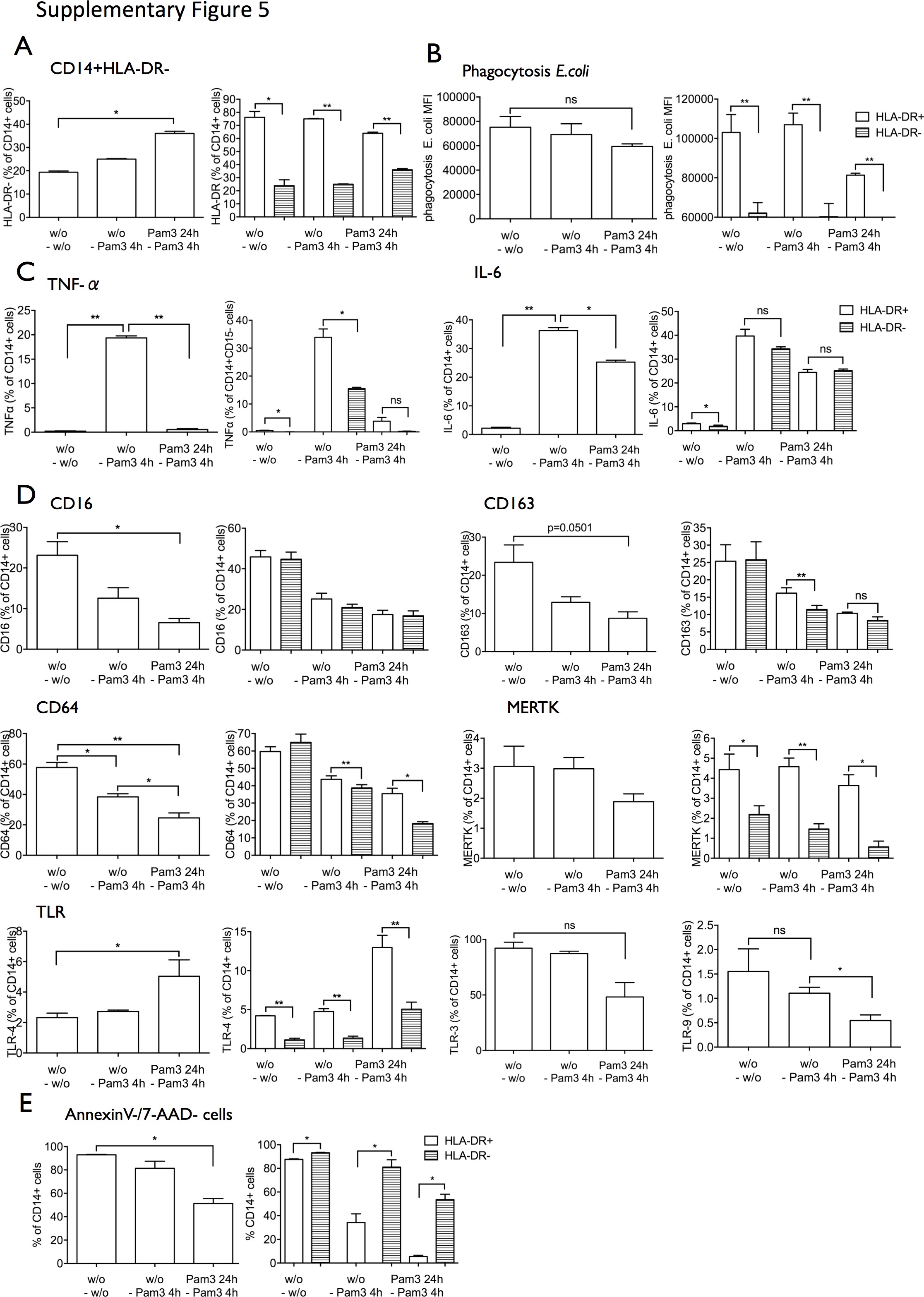

Supplement: Supplementary Figure 5 [file gutjnl-2017-314184supp005.jpg]
